# Advancing energy integration: renewable sources, ancillary services, and stability

**DOI:** 10.1371/journal.pone.0324812

**Published:** 2025-06-02

**Authors:** Asif Gulraiz, Syed Sajjad Haider Zaidi, Bilal Mohammad Khan

**Affiliations:** 1 Electrical & Power Engineering Department, National University of Science & Technology (PNEC) , Karachi, Sindh, Pakistan; 2 Electrical Engineering Department, DHA Suffa University, Karachi, Sindh, Pakistan; Joginpally B R Engineering College, INDIA

## Abstract

The paper discusses the recent developments and challenges in the energy sector, particularly focusing on the integration of renewable energy sources into microgrids and conventional power systems. It highlights the importance of predicting future energy generation for effective grid integration and discusses the use of Artificial Neural Network (ANN) and Auto-Regressive Moving Average (ARMA) models for this purpose. Additionally, it explores the role of distributed generation in providing ancillary services traditionally offered by conventional power systems and analyzes the impact of renewable energy sources on core parameters like frequency and voltage stability. It also discusses the rapid growth of solar photovoltaic (PV) systems and the need to assess their impacts on distribution networks. Furthermore, it addresses the ongoing energy crisis, particularly in South Asia, and proposes solutions such as power factor correction through technologies like Static VAR Compensators (SVCs) to enhance system stability and efficiency, especially in medium and long transmission lines.

## 1 Introduction

Renewable energy integration presents both opportunities and challenges for power systems worldwide. To address these challenges, adjustments to conventional grids are necessary, including managing generator output and forecasting renewable energy consumption. Energy modelling informs policy decisions and infrastructure development, aiding efficient energy supply. In Saudi Arabia, ambitious renewable energy goals highlight the importance of understanding the impacts of integration on system stability. Different Wind Turbine Generators (WTG) and Photovoltaic (PV) systems influence system behaviour differently, necessitating thorough analysis using tools like the IEEE 30 bus power network and ETAP software. This research underscores the need to mitigate potential destabilizing effects of renewable integration.

Driven by climate change concerns, the shift to renewable energy is imperative. However, power grid instability persists due to generation and transmission disruptions. Robust controllers and network improvements are essential for stability management, considering factors like generator loading and system impedances. In Pakistan, increasing reliance on renewables aligns with sustainable development goals (SDGs). Solar energy offer decentralized electricity generation opportunities, but challenges like voltage level fluctuations arise with increased PV integration. Evaluations using Cyme software analyse scenarios to optimize renewable energy integration in Pakistan’s electricity infrastructure. Transmission systems face strain from rising demand, necessitating innovative solutions like Static VAR Compensators (SVCs). Leveraging approaches like bare metal coding optimizes SVCs performance, enhancing grid stability and efficiency. This research contributes valuable insights into resilient, sustainable power systems, addressing operating principles, methodology, hardware description, results, and conclusions comprehensively.

### 1.1 Motivation

Energy forecasting is considered to be an important aspect while considering Renewable Energy Generation (REG). For forecasting energy demand, the GARGM method enhances the GM model with a genetic algorithm for improved accuracy using data from China [1]. A framework integrating climate factors with energy-economy models shows residential energy demand is more sensitive to climate changes than commercial demand [2]. Achieving 100% REG in South and Central America by 2030 is possible using hydropower, wind, and solar power, with hydroelectric dams acting as virtual batteries [3]. New controller designs (JBO and BA) and optimization methods Smell Agent Symbiosis Organism Search Algorithm(SASOS) enhance microgrid frequency stability and control [4–6]. Machine learning helps identify power quality events in REG based microgrids [7]. Combining dynamic voltage restrorer (DVR) and fault current limiter (FCL) improves wind turbine performance during voltage dips [8]. ANFIS models combined with particle swarm optimization (PSO) or Genetic algorithm (GA) accurately predict wind power density in Malaysia [9]. A method optimizing active power output of wind farms enhances grid integration using data from Bangladesh [10]. A control strategy (I+ECO+V2G) significantly improves electricity frequency stability in systems with high renewables and electric vehicles (EVs) [11]. Large-scale solar power integration can reduce grid stability, necessitating careful management [12]. In Amherst, Massachusetts, Bidirectional Long Short-Term Memory (Bi-LSTM) models provide the best accuracy for solar power predictions [13].

Providing reliable electricity for islands and remote areas is addressed by hybrid systems combining solar, wind, and battery storage [14]. The ACT method accurately estimates solar cell properties for better performance simulations [14]. A generalized global sliding mode controller (GGSMC) with feed-forward neural network (FFNN) effectively extracts maximum power from PV systems [15]. A new control strategy using PSO fine-tunes a proportional integral controller (PI) controller, improving power quality for grid-connected solar PV systems [16]. An adaptive fuzzy sliding mode controller enhances the efficiency and reliability of solar power systems [17]. Installing solar panels on residential buildings in Jeddah, Saudi Arabia, shows significant potential for meeting energy needs and reducing emissions [18]. Analysing a 10-megawatt solar plant in Iran provides insights for solar power investors [19]. Polycrystalline silicon panels outperform others in Morocco, with accurate power predictions [20]. Identifying buildings for rooftop solar in Bandung, Indonesia, helps promote clean energy use [21]. Solar panels in Dublin, Ireland, reduce emissions and offer competitive costs with industry support [22]. University buildings offer significant potential for solar energy use [23,24]. Optimizing SVC placement enhances grid stability and reduces costs [25]. A nature-inspired algorithm optimizes solar plants and SVCs, minimizing power losses and improving resource use [26]. Wind speed prediction is crucial for maintaining stable power grids with significant wind energy contributions. A new method combining MLP, KNR, and LSTM models, optimized by an ADGWDTO algorithm, outperforms previous methods in accuracy [27].

### 1.2 Paper contribution

This paper contribution is divided into different parts as it covers different aspect of multiple energy types. Following are the contribution of this paper.

- Renewable Energy prediction using ANN is covered in this literature using real data from EU countries.
- Micro Grid stability assessment is done IEEE 30 bus system in the presence of Wind turbine and Photovoltaic generation.
- Transient stability analysis is performed using Type-III Wind turbine for comparative analysis with conventional generators for low-voltage ride through (LVRT) and high voltage ride through (HVRT) response.
- Photovoltaic assessment in the presence of smart inverter controls is covered using real time bus data from a low voltage network of the city.
- SVCs for medium transmission lines are designed and it is tested on the indigenous designed medium transmission line trainer.

### 1.3 Paper formation

The formation of the remaining paper is as follows. In Section 1, a detailed introduction is presented covering motivation, paper contribution and paper formation. Literature review is covered in Section 2 covering all the aspects which are considered essential for this topic are discussed. In Section 3, methodology schemes are discussed. Simulation results are covered in Section 4. Section 5 concludes this paper with recommendations and suggestion for future distributed generation networks.

## 2 Literature review

### 2.1 Artificial neural network

**Fig 1:**
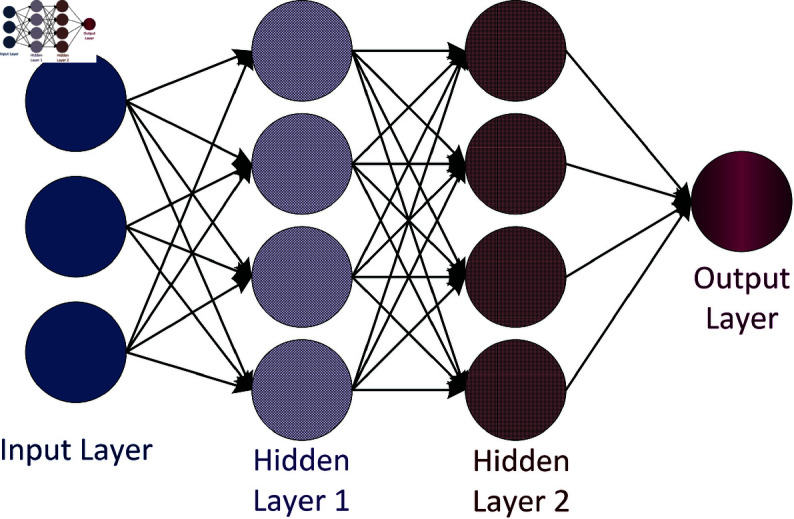
ANN structure.

Load forecasting is done using regression analysis [28–31] but REG prediction based on ANN offers a promising modelling approach, simulating human brain behaviour by aggregating artificial neurons into layers as shown in Fig 1. These layers are trained to resemble target data sets, with signals passing from input to output layers through hidden layers. ANN transcends analytical limitations, efficiently tackling complex modelling issues with the ability to learn from data samples. Particularly useful for non-linear problems like energy forecasting, ANN finds applications across various sectors, from finance to biomedical fields. In contrast, Autoregressive Moving Average (ARMA) models, such as ARMA(p,q), are suitable for univariate time series modelling, representing future variable values as linear combinations of past observations with random errors.

In the past, load forecasting primarily relied on regression analysis, as evidenced by referenced papers [28–31] which focus on load or renewable energy consumption forecasting. One study [28] presents pragmatic approaches to energy demand forecasting, utilizing electrical Power Load models based on load time series disintegration, using data from the Kuwaiti Electricity network. Another paper [29] explores support vector machines (SVM) for time series prediction, with a focus on load forecasting. Monthly electricity consumption forecasting is addressed in [30], employing feature extraction techniques like Principal Component Analysis (PCA). Additionally, some researchers [31] utilize PCA for weather condition forecasting. Historically, renewable energy forecasting was not prioritized due to limited renewable resource utilization. However, as reliance on renewable energy increases, there’s a paradigm shift towards considering renewable energy as a reliable grid resource. Hence, there’s a growing need for accurate renewable energy forecasting to ensure grid reliability. Another paper [1] focus on Remnant gray prediction model for energy forecasting. Since climate change has its impact on energy demands and generation type [2], deep learning methods and ANN are used widely for PV energy predictions [32–34].

### 2.2 Transitioning to sustainable power: addressing grid stability

Climate change is a pressing issue caused by greenhouse gas emissions, particularly from power plants using coal, natural gas, oil, and nuclear energy [35]. These emit significant amounts of CO_2_, exacerbating global warming [36]. To address this, sustainable power projects like WTGs and PVs are vital for clean energy generation. Power grid instability, caused by disturbances in generation, transmission, and distribution units, poses challenges. Stability in the power system is crucial for ensuring uninterrupted supply, with factors such as the weight of rotating parts in generators, system impedances, fault duration, generator loading, and overall system loading influencing stability [37]. Addressing these factors is essential for maintaining a stable power grid, several studies are conducted to for transient stability assessment of different parameters considering WTGs, PV and conventional generators (CGS)[38,39]. Frequency stability is also widely assessed in the presence of Renewable generation as they will offer better stability as in [3–6]. Power quality is also an important aspect while considering transient studies as WTGs and PV can offer better power factor as compare to CGS (CGS) [7].

### 2.3 Navigating renewable integration: challenges and innovations in power systems

Alternate Energy plays a pivotal role in driving industrial progress and societal advancement. It acknowledges the gradual shift from conventional energy sources to renewable ones, aiming to tackle energy crises. Middle East country’s ambitious renewable energy goal and the global surge in renewable generation capacity underscore this shift. However, integrating wind and solar power presents challenges to power system stability, particularly during faults [40]. The analysis emphasizes the importance of transient stability assessment, focusing on voltage regulation, active power input, and reactive power capabilities. Wind turbine generators, especially Type 3, enhance stability through reactive power compensation, while studies explore fault tolerance in wind turbines [41]. A research article [42] discusses fault tolerance in wind turbines, proposing strategies like Fault-Tolerant Individual Pitch Control (FTIPC) to mitigate emergency shutdowns and maintenance costs. The study suggests a novel approach using linear approximating parameters and interconnected control units to enhance fault tolerance and overall turbine performance. HVRT and LVRT control capability offers by WTGs are better as evident from [8,9,43]. In [10,11] power system control strategy is widely discussed showing potential of WTGs penetration in existing power system, while in [12,44] in the presence of PV system power system stability is covered.

### 2.4 Pakistan’s transition to renewable energy

Pakistan, as a developing nation, is increasingly focusing on renewable energy for electricity generation. This shift aligns with the United Nations SDGs and allows people not only to power their homes but also to sell excess electricity to local power companies [45], contributing to the renewable energy transition. Solar panels, particularly photovoltaic systems, are popular for direct sunlight conversion into electrical energy, enabling individuals to become electricity producers. Net metering facilitates this process, allowing consumers to sell surplus electricity back to utility companies, offsetting their investment costs [46–49]. However, increased penetration of photovoltaic systems in low-voltage distribution networks raises voltage levels, presenting a challenge [13,18,19,47]. In [14–17] different techniques are also applied to improve the PV output for stand-alone systems and hybrid PV systems specifically for low voltage networks. Rooftop PV assessment is required to find the impact of PV system on grid voltages due to dynamic weather conditions as it is done in [21–23]. In this paper an analysis of the existing distribution network in Karachi is conducted, different scenarios of photovoltaic insolation levels due to weather conditions are evaluated using the Cyme software, a power engineering tool for analysing generation, transmission, and distribution networks. This analysis helps understand the impact of varying photovoltaic penetration on the overall system, informing strategies for integrating REG into distribution systems effectively.

### 2.5 The role of static VAR compensators

In the face of increasing demand, transmission systems are facing different issues due to losses occurred in medium and long transmission system it also deterred the power quality of the system as various paper covers methods to improve power quality of transmission system based on SVCs [25,26,50]. Transmission lines often operating below their thermal rating due to stability limits and low power factor issues [51]. To address these challenges, SVCs have emerged as crucial solutions, managing reactive power within electrical power systems. SVCs, composed of capacitors, switches, and a microcontroller, strategically adjust reactive power based on system needs. Microcontroller-based automatic power factor compensation using capacitor bank switching offers a viable solution. Capacitor switching, activating or deactivating capacitors within the SVCs [52], generates or absorbs leading reactive power to compensate for system lag. While SVC technology is mature, ongoing research explores innovations like bare metal coding for optimized performance, promising enhanced power system stability. Leveraging SVCs to their full potential, combined with innovative techniques, can minimize voltage fluctuations, reduce power losses, and enhance overall grid efficiency [53]. Such advancements contribute to resilient, intelligent, and sustainable power systems, meeting the demands of our modern world. This paper probes into SVCs technology, covering principles, operating mechanisms, and potential advancements.

## 3 Methodology

This paper covers five different areas of work which include renewable energy prediction based on ANN, addressing grid stability issues using a sample IEEE 30 bus system, Renewable energy integration in the existing power system, Pakistan’s transition to renewable energy and role of SVCs in optimizing transmission line operation.

### 3.1 Renewable energy prediction based on ANN

**Fig 2:**
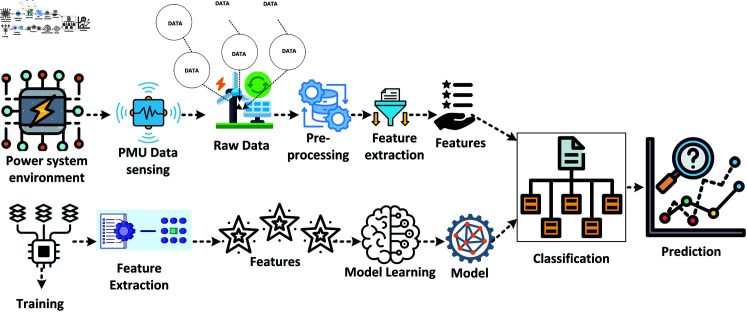
Process diagram for energy prediction.

This section covers renewable energy forecasting in alignment with the UK’s 2020 and 2030 targets. The prediction process, illustrated in Fig 2, involves an experimental and validation study that includes collecting PMU data, processing missing values, and selecting an ARMA model based on ANN techniques. Data taken for this study is from the actual network located in Europe. The data is split into training and testing sets, with three years of data used for training and one year reserved for validation. The results are plotted to compare actual versus predicted data, ensuring the robustness and accuracy of the forecasting model.

### 3.2 MicroGrid stability analysis

**Fig 3:**
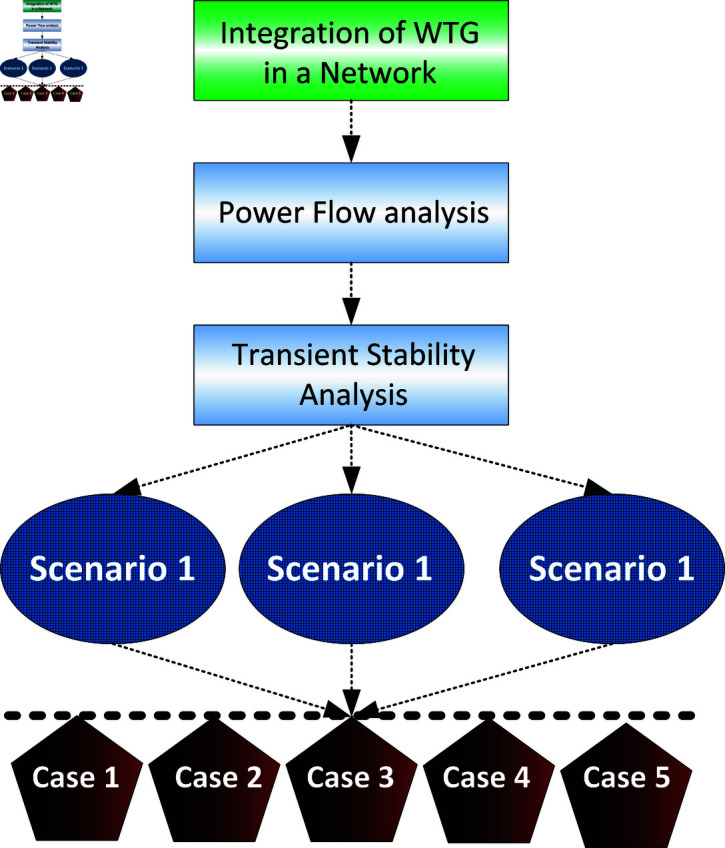
Transient stability analysis framework for micro grid.

**Fig 4:**
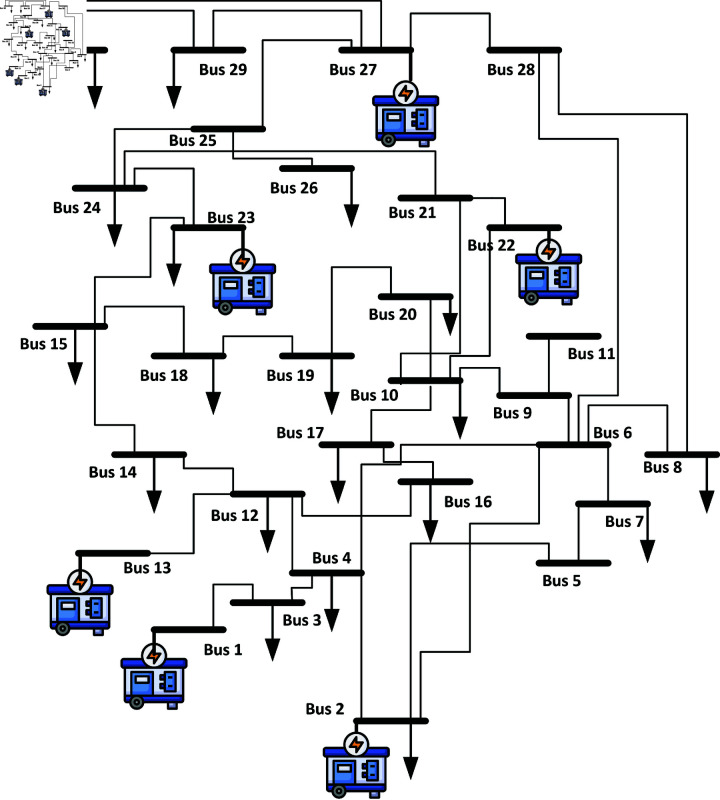
IEEE 30 bus system.

In this section microgrid system is discussed the support for grid through Wind turbine Generators (WTGs) and Photovoltaic (PV) are covered to address grid stability issues. Simulations were conducted on an IEEE 30 Bus system as shown in Fig 4, with the 132 kV bus 13_8 selected as the connection point for PV and WTG, given its significant generation capacity of 90 MW. Three different scenarios were examined to assess transient stability within the microgrid as shown in Fig 3. In Scenario 1, a three-phase fault was introduced between buses 13.8 and 13_6, analysing the voltage profile and phase at the faulted bus and surrounding buses. Scenario 2 involved a 150 percent load impact on Load 8, with the analysis focusing on the voltage and frequency of bus 13_8 and neighbouring buses. Scenario 3 simulated a three-phase fault on swing bus 13_1, analysing the frequency of the faulted bus and its impact on adjacent buses. Each scenario was simulated with synchronous generators, PV arrays, and WTG iteratively connected to the coupling point. Voltage stability, a persistent concern in power systems, arises when disturbances like faults or load changes occur, leading to inadequate reactive power and voltage drops below acceptable levels. This instability can trigger the tripping of transmission lines, representing a long-standing challenge for power industries caused by mismatches in real power, affecting the speed of generating units [33,34].

### 3.3 Transient stability analysis on wind turbine integration

Transient stability in power systems is essential for maintaining voltage levels during disturbances, whether caused by sudden demand spikes or generator fluctuations. Voltage stability ensures acceptable levels across all buses, managed through HVRT and LVRT techniques. HVRT enables turbines to withstand over-voltage conditions, while LVRT allows them to tolerate voltage drops. This part focuses on equipping synchronous and wind turbine generators with voltage controllers. AVR_IEEET1 regulates synchronous generators, while wind turbines utilize fast local voltage controllers to adjust KVAR loading for stable voltage, especially during short circuits. Frequency stability is critical for maintaining balance between generation and load. Power electronic speed controllers for both generator types are examined to prevent rotor speed destabilization, which can occur during load fluctuations. Power System Stabilizers (PSS) enhance system damping oscillations and stability by controlling parameters like shaft speed and terminal frequency. These measures ensure a stable power supply despite disturbances in the system. The IEEE 30 bus system is used for the above defined analysis as shown in Fig 4.

### 3.4 Roof top photovoltaic assessment on low voltage network

**Fig 5:**
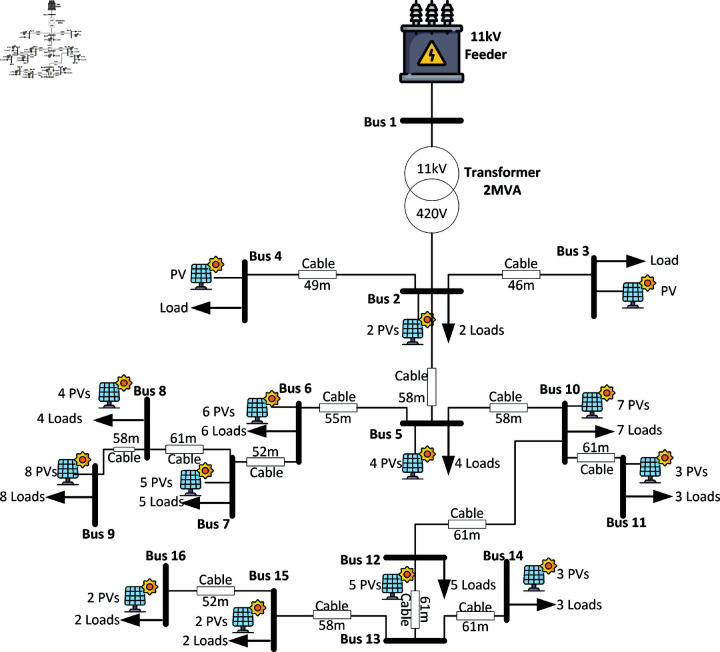
Unbalanced load based distribution system.

**Fig 6:**
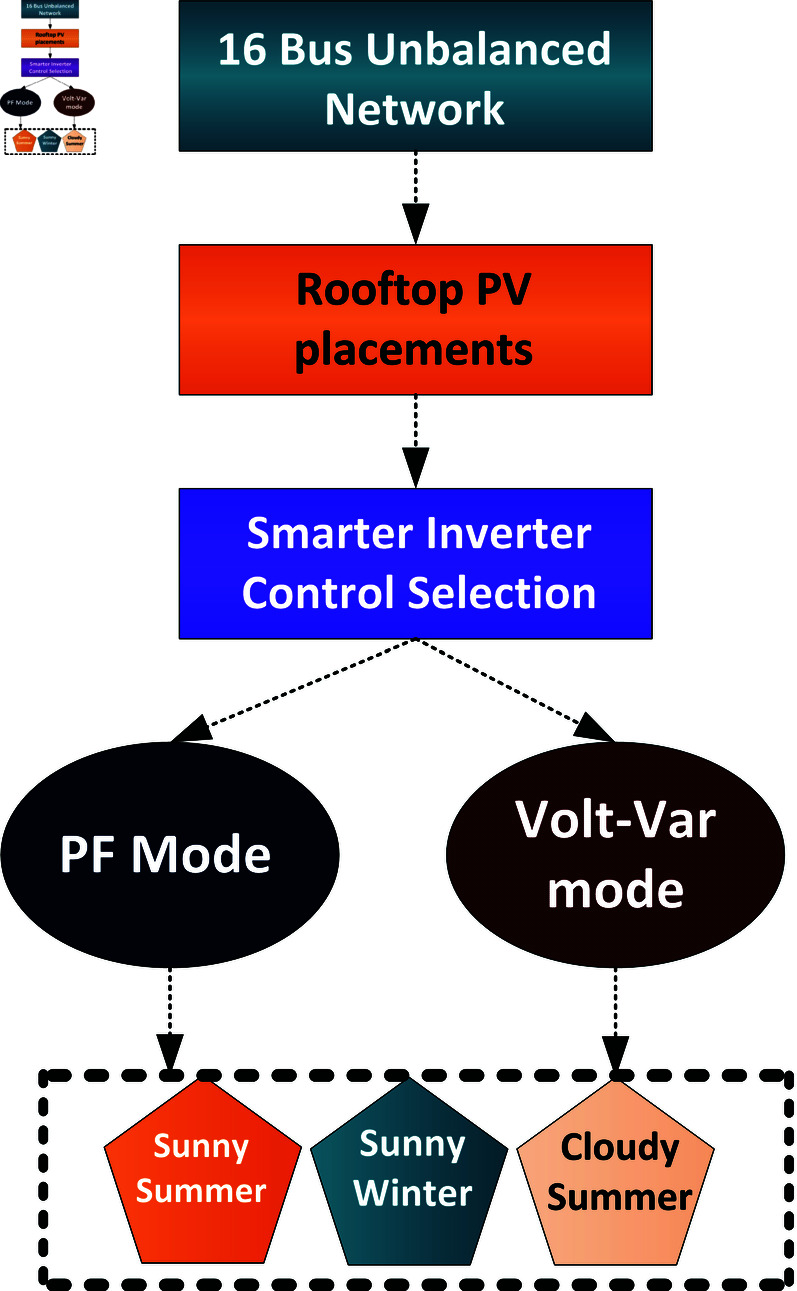
Simulation scenarios of smart inverter control system.

A representative network model, designed to replicate Pakistan’s power network at distribution level. It was developed using CYME software as shown in Fig 5 with parameters detailed in Table 1, Cyme is a professional software use by the utility company in Pakistan and it is also use by other utility companies all over the globe. The network, which serves as a representation of an actual distribution system, cannot disclose its name or location due to restrictions imposed by the utility company. The unbalanced network in Fig 5 illustrates a feeder comprising a transformer and 16 buses, connecting loads and PV generation units to analyse the impact of rooftop PV systems integrated with net metering. Simulations were conducted under two inverter control modes: Power Factor (PF) and Volt-Var control, with high PV penetration levels exceeding 150%. Three distinct cases (Fig 6) were examined: Case A (sunny summer conditions) with high demand (1-4 kW per load), Case B (sunny winter conditions) with reduced demand (1 kW per load), and Case C (cloudy summer conditions) featuring variable PV generation and a load of 2 kW per residence. Voltage control strategies included PF control for optimal efficiency (power factor = 1) and Volt-Var control to mitigate voltage fluctuations. Additionally, the location of PV systems was found to influence short-circuit currents, with proximity to the feeder being a key factor.

**Table 1:** Scenarios of microgrid transient analysis.

S.no.	Bus #	Loads connected	Phases
1	2	2	A,B
2	3	1	C
3	4	1	A
4	5	4	B, A, B, C
5	6	6	A, B, C, A, B, C
6	7	7	A, B, C, A, B, C, C
7	8	5	A, B, C, A, B
8	9	4	C, A, B, C
9	10	8	A, A, B, C, A
10	11	3	A, B, C
11	12	5	C, A, B, C, A
12	13	4	B, A, B, C
13	14	3	A, B, C
14	15	2	C, A
15	16	2	B, C

### 3.5 Role of static VAR compensators (SVCs) in medium transmission line

**Fig 7:**
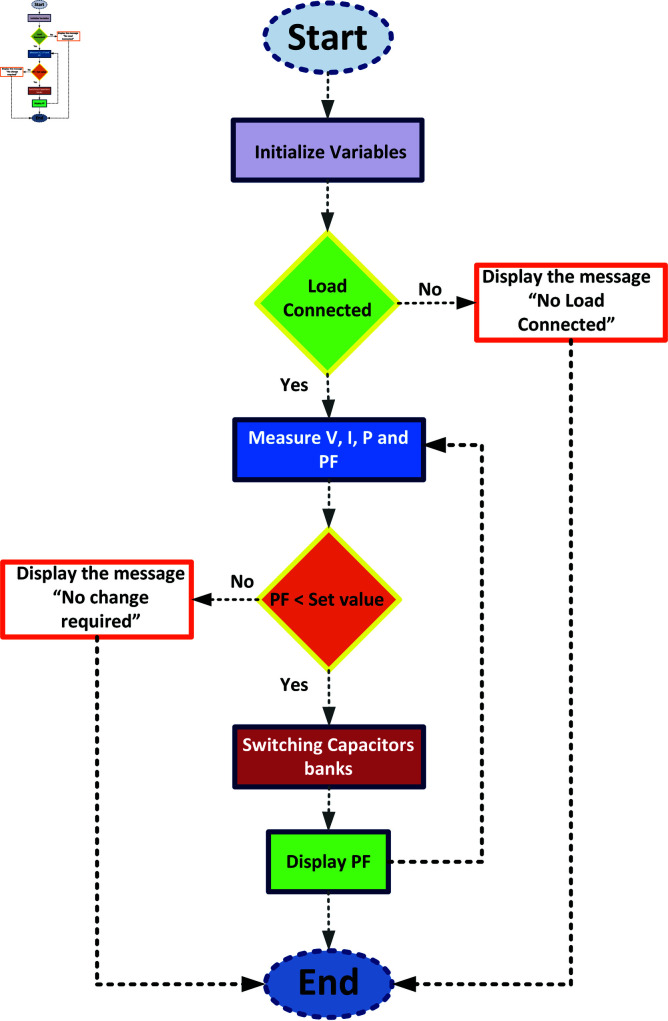
Flow chart for SVC switching.

This study uses a Hardware in the loop (HIL) system based on De Lorenzo transmission line trainer, Arduino microcontroller, PZEM-004T sensor, relay module, and capacitor bank to evaluate a Static VAR Compensator’s (SVC) effectiveness in improving power factor. A medium-length transmission line is emulated, integrated with the PZEM-004T energy meter and Arduino to monitor parameters and trigger capacitor bank connection via a relay module. In Fig 7, framework for SVC switching is shown.

## 4 Simulation results and discussion

### 4.1 Renewable energy prediction based on ANN

The prediction data package includes time series data on electricity consumption (load), wind energy (generation & capacity), PV energy (generation & capacity) in quarter-hourly, half-hourly, and hourly resolutions. Aggregated by country, it spans EU nations from 2015 to 2020.

#### 4.1.1 Feature extraction

**Fig 8:**
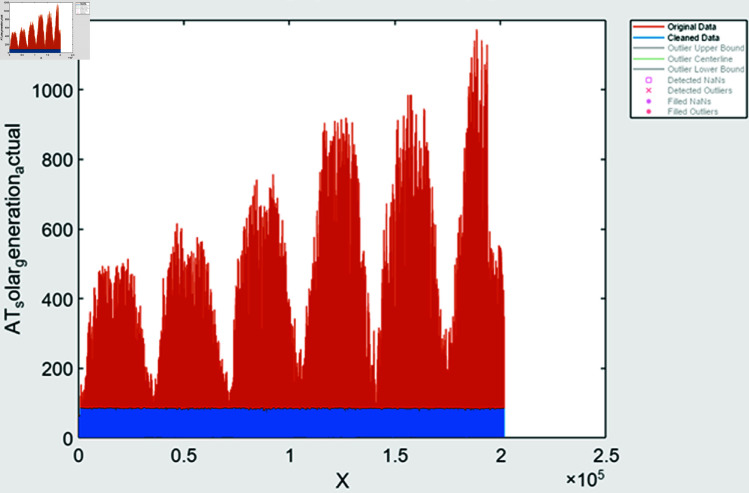
Solar generation Austria data after feature extraction.

**Fig 9:**
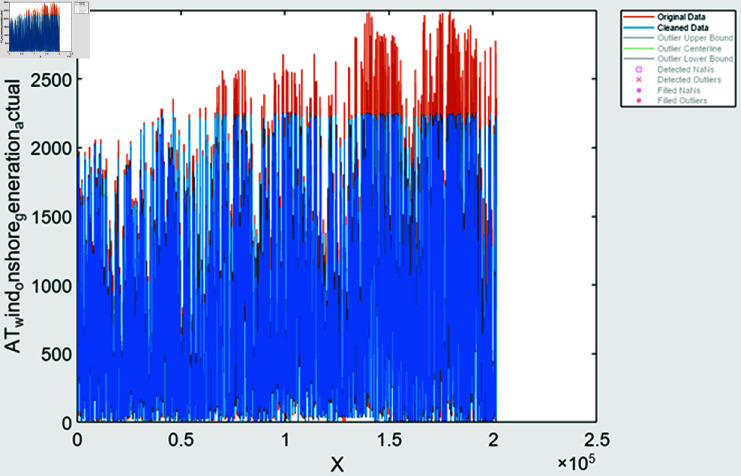
Wind generation Austria after feature extraction.

**Fig 10:**
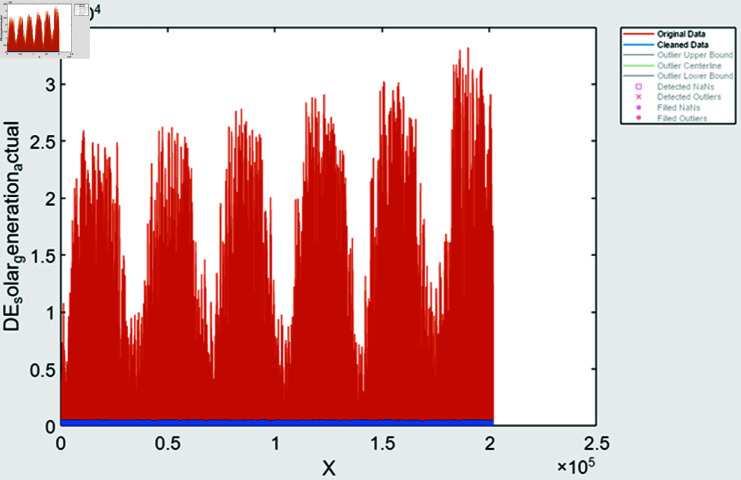
Solar generation Germany after feature extraction.

**Fig 11:**
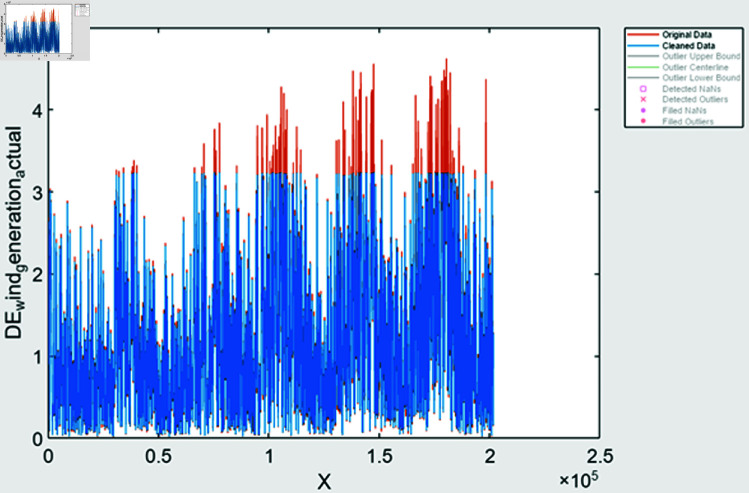
Wind generation Germany after feature extraction.

During time series extraction, the raw data often contains outliers and inaccuracies due to missing values and human errors. To address this, various methods are employed; it includes missing value handling methods it identifies missing values and filled based on trends observed in the data, outlier detection is done using statistical techniques to identify and fill outliers in the data, ensuring more accurate representation, data smoothing is also done to remove noisy data. After performing feature extraction, the data is visualized to assess the effectiveness of the processing steps. In Figs 8 and 9, solar and wind energy data of Austria is displayed post-processing, with missing values filled using the “fill outliers” command. Similarly, Figs 10 and 11 illustrate Solar and Wind generation data, highlighting the presence of missing values. Addressing these gaps and ensuring data accuracy is crucial for accurate prediction and analysis of future trends.

#### 4.1.2 Feature classification

ANN classification is a prime example of supervised learning, where known class labels are utilized to verify system accuracy or guide its performance. The Feed-forward (FF) network architecture is commonly employed in ANN, comprising multiple layers where each layer receives connections from the previous one, ultimately producing the network output.

**Fig 12:**
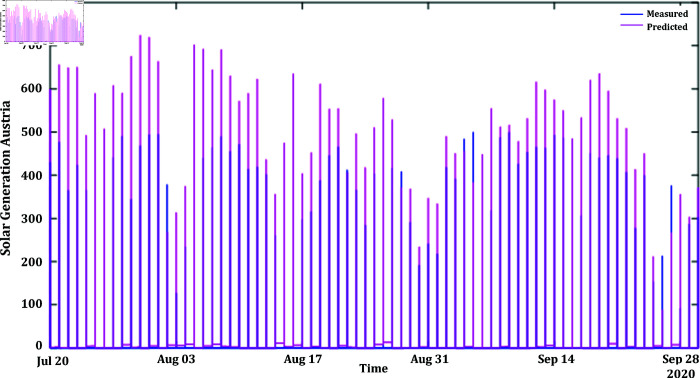
Solar energy Austria actual vs predicted after feature classification.

**Fig 13:**
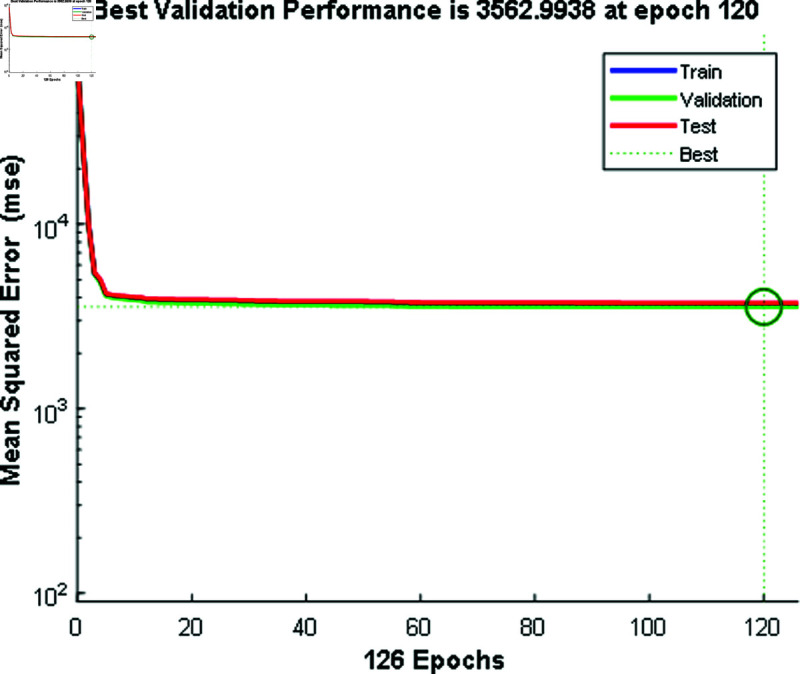
Performance graph for solar energy Austria.

**Fig 14:**
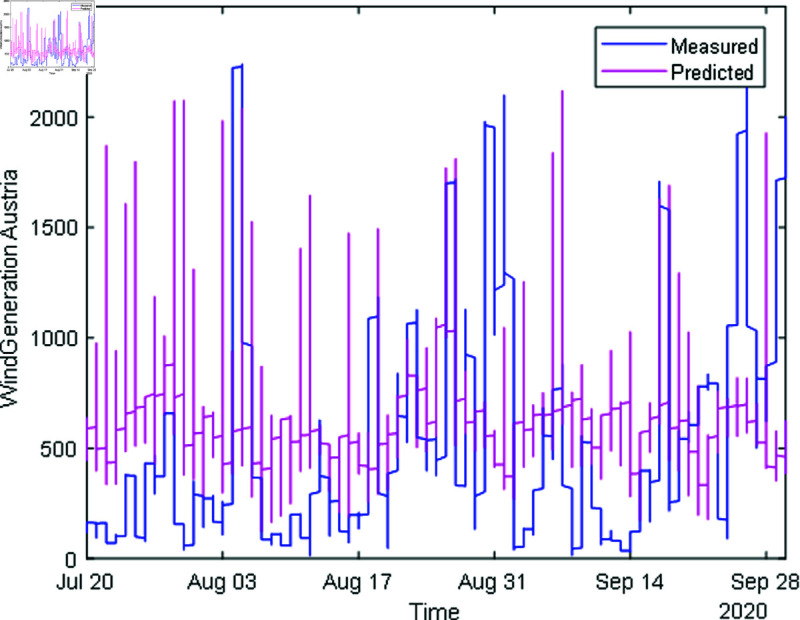
Wind energy generation Austria after feature classification.

**Fig 15:**
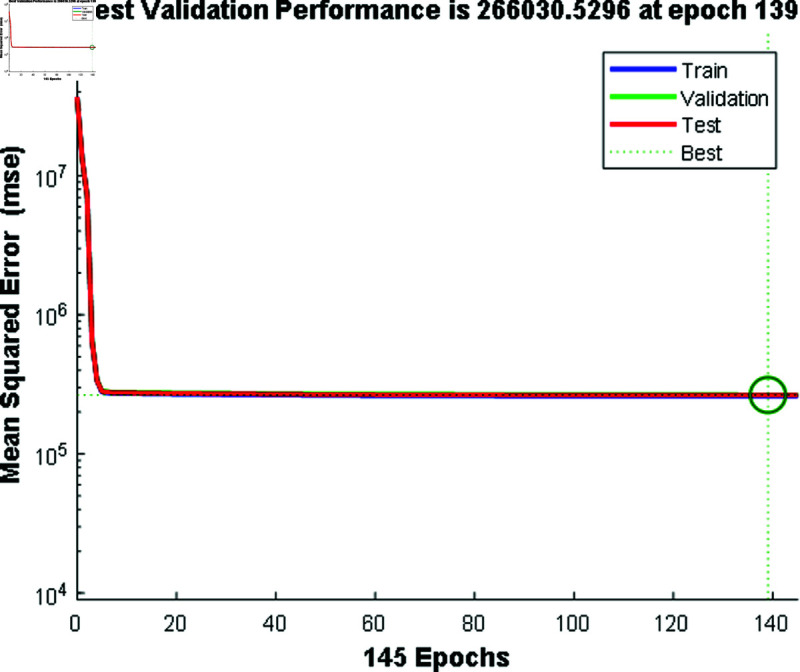
Performance graph for wind energy Austria.

**Fig 16:**
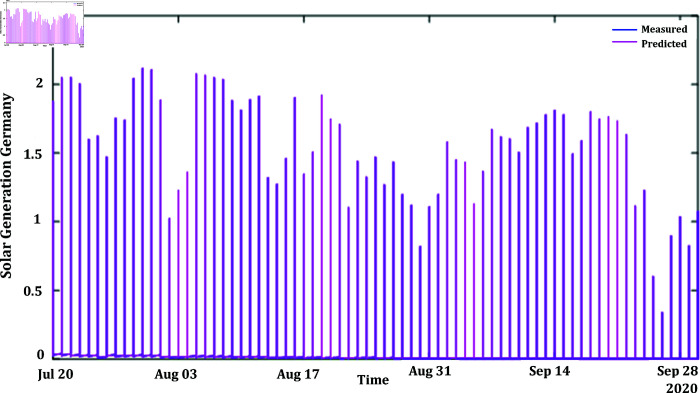
Solar generation Germany after feature classification.

**Fig 17:**
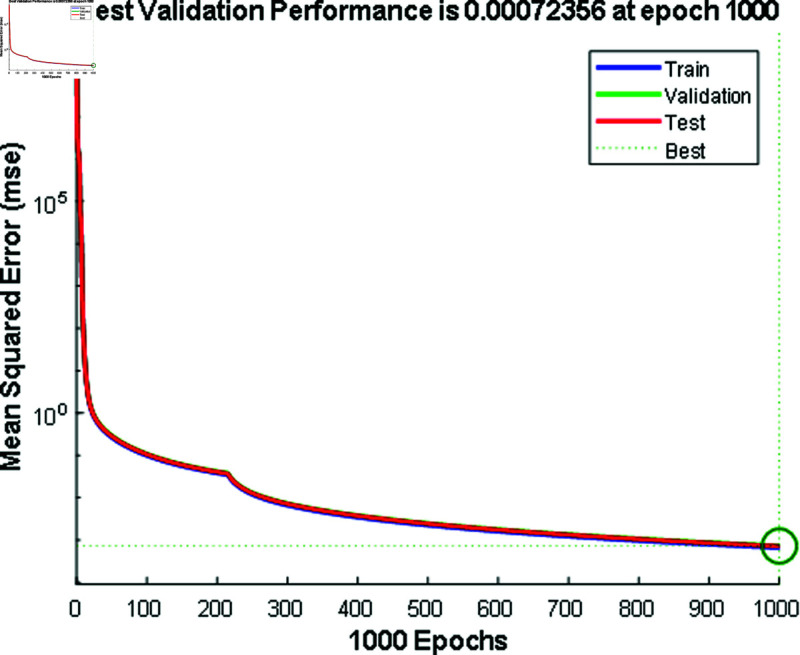
Performance graph for solar energy Germany.

**Fig 18:**
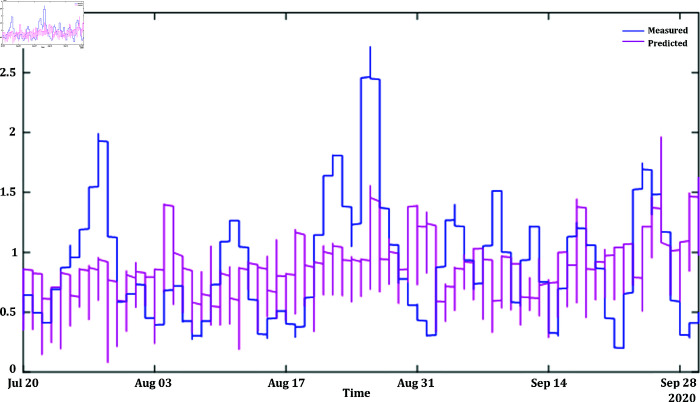
Wind energy generation Germany after feature classification.

**Fig 19:**
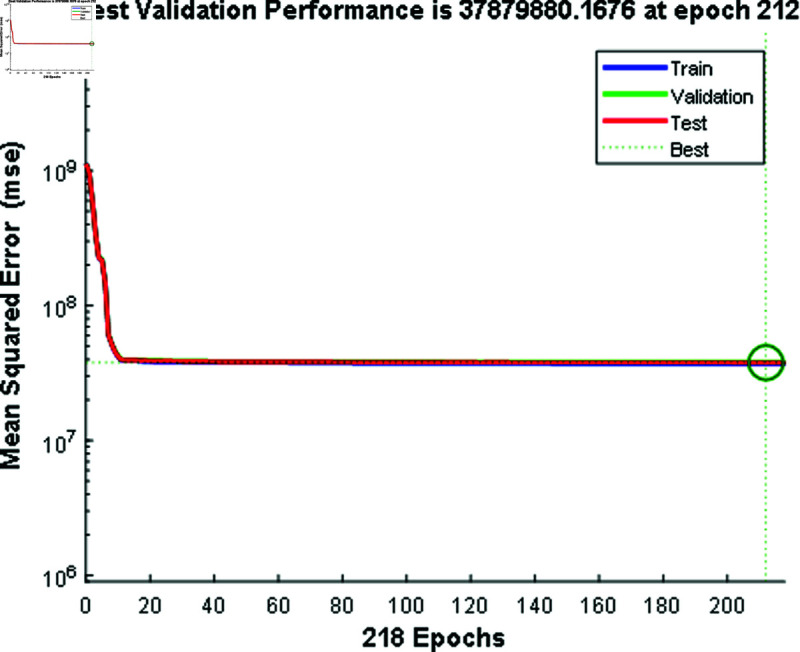
Performance graph for wind energy Germany.

In Figs 12 and 14, the predicted values for solar and wind closely align with the measured values, confirming the algorithm’s functionality. Figs 13 and 15 displays the learning curve for Solar and wind energy prediction in Austria, showcasing the convergence of predicted and actual data. Similarly, In Figs 16 and 18 validates Wind and solar energy data for Germany, with minor disparities between predicted and measured values. An optimal learning curve is depicted in Figs 17 and 19 for renewable energy prediction in Germany.

### 4.2 Microgrid stability analysis on IEEE 30 bus system

Simulations on a modified IEEE 30 Bus system selected bus 13_8 (132 kV) for integrating PV and wind turbine systems due to its 80 MW generation capacity. Three scenarios were analysed as shown in Table 2 to assess transient stability in the microgrid, involving cases for varying PV and WTG penetration levels compared to a conventional generator system (CGS) as mentioned in Table 3.

**Table 2:** Scenarios of microgrid transient analysis.

Scenarios	Description
Scenario 1	The transmission line between bus 13_8 and 13_6 experiences a three-phase fault.
Scenario 2	Load 8 experiences an impact, increasing to 150 percent of its nominal value.
Scenario 3	A three-phase fault is simulated on the swing bus 13_1.

**Table 3:** Scenarios of microgrid transient analysis.

	Synchronous Generator	Wind Turbine Generator	Photovoltaic
Case-1	100%	–	–
Case-2	50%	50%	–
Case-3	25%	75%	–
Case-4	50%	–	50%
Case-5	25%	–	75%

#### 4.2.1 Scenario 1

**Fig 20:**
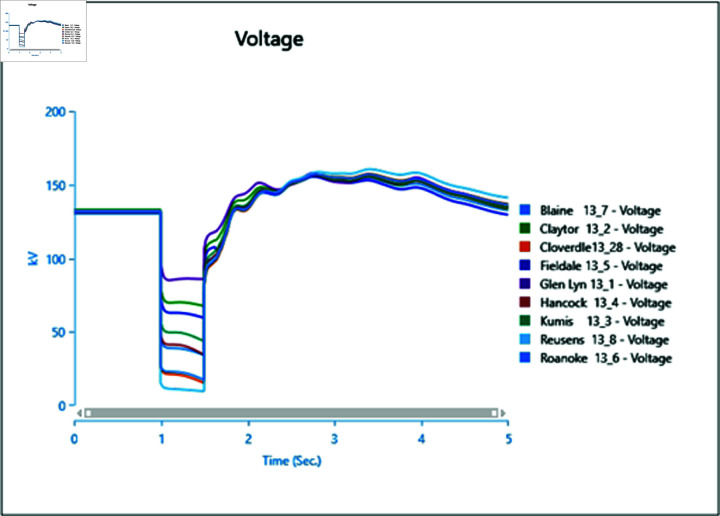
Case 1: scenario 1 (voltages).

In the first scenario outlined in Table 2, a three-phase fault is initiated at bus 13_8 at 1.0 second and rectified at 1.5 seconds. The simulation extends over a total runtime of 5 seconds, during which bus voltages and voltage angles are scrutinized for a duration of 0.5 seconds. Plots depicting the analysis results for cases 1 through 5 are presented sequentially in Fig 20 to Fig 30.

**Fig 21:**
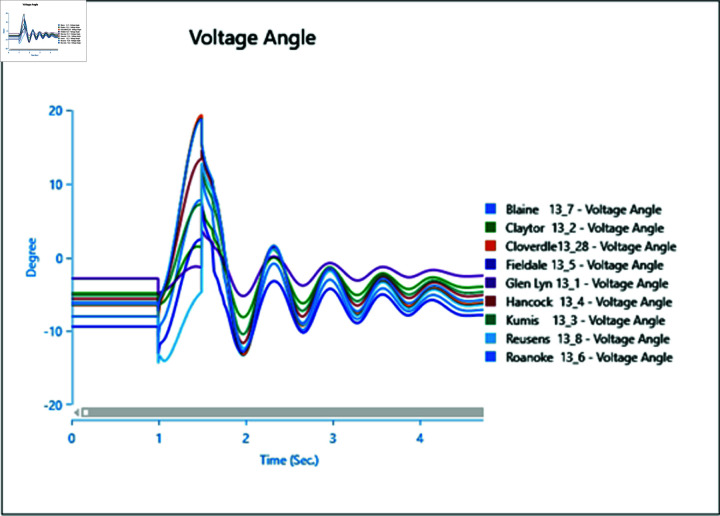
Case 1: Scenario 1 (voltage angle).

**Fig 22:**
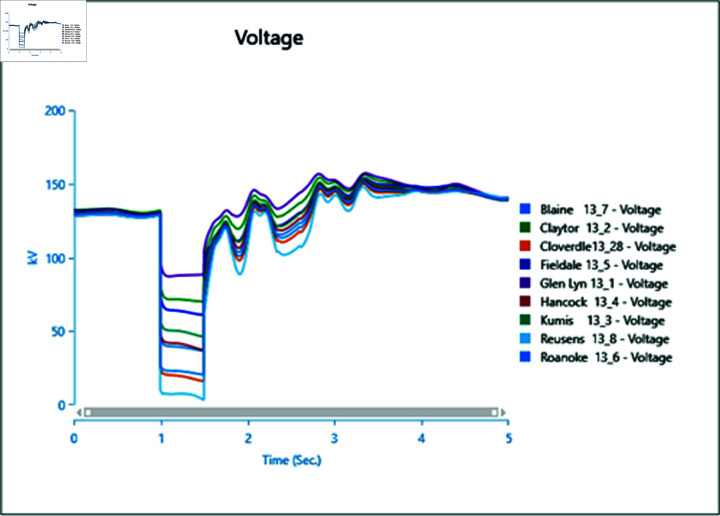
Case 2: Scenario 1 (Voltages).

**Fig 23:**
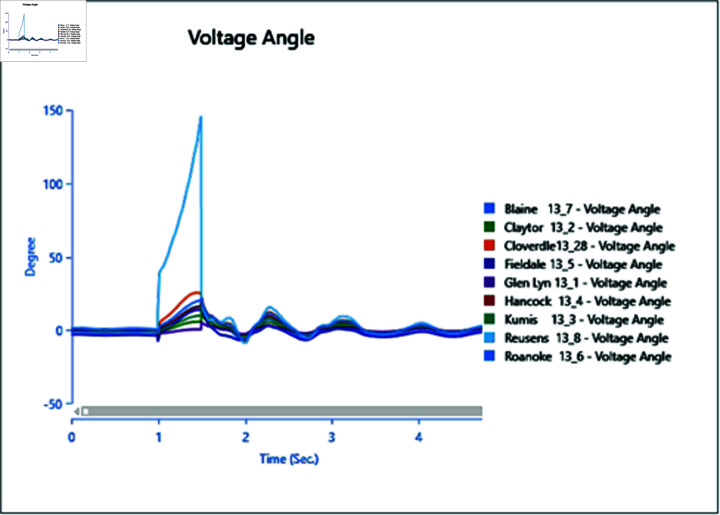
Case 2: Scenario 1 (Voltage angle).

**Fig 24:**
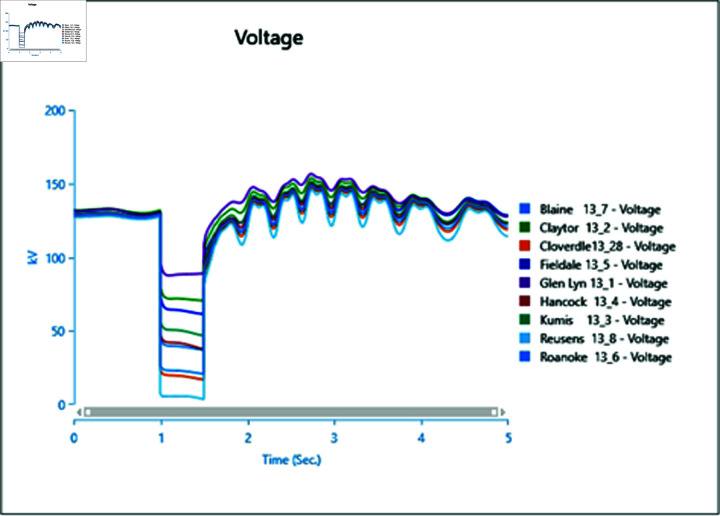
Case 3: Scenario 1 (voltages).

**Fig 25:**
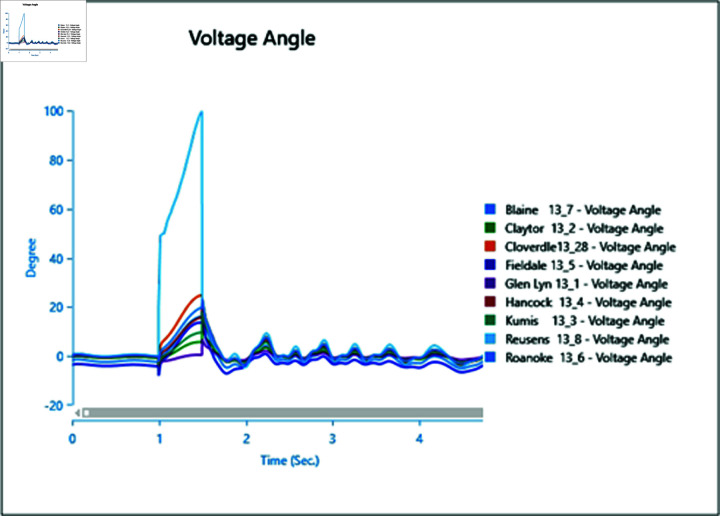
Case 3: Scenario 1 (voltage angle).

**Fig 26:**
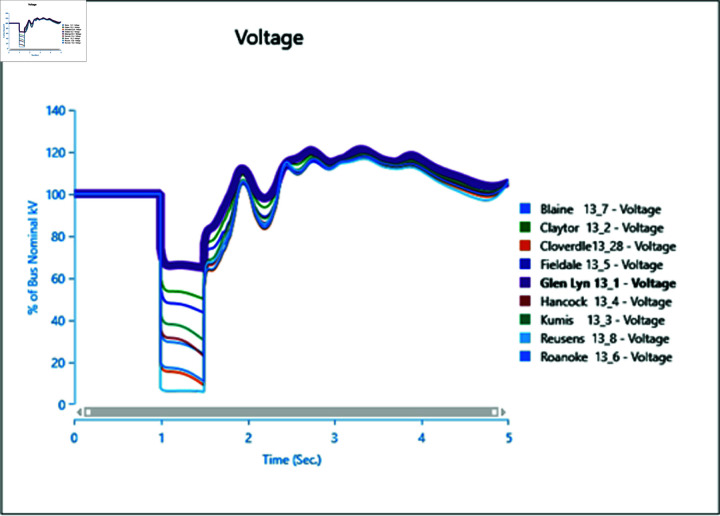
Case 4: Scenario 1 (voltages).

**Fig 27:**
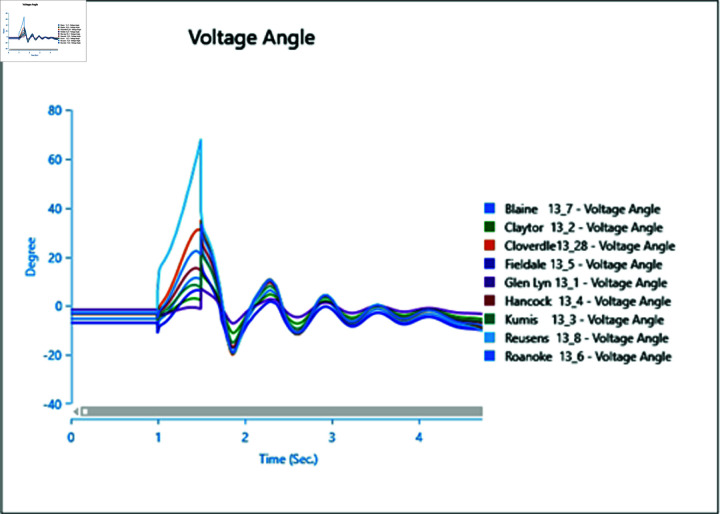
Case 4: Scenario 1 (voltage angle).

**Fig 28:**
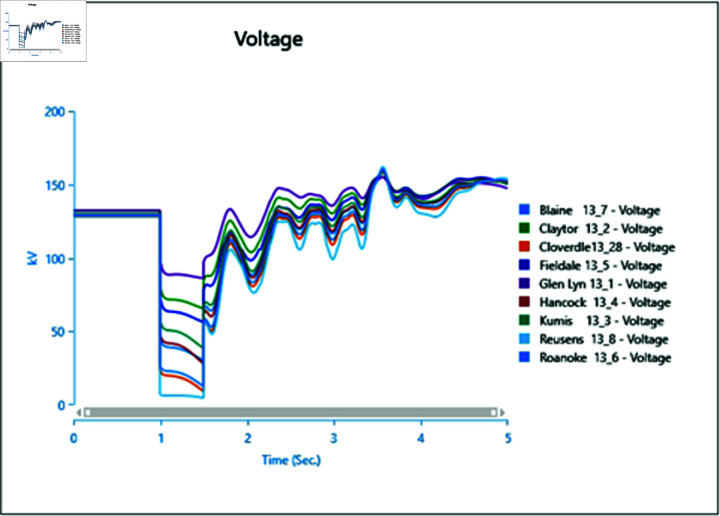
Case 5: Scenario 1 (Voltages).

**Fig 29:**
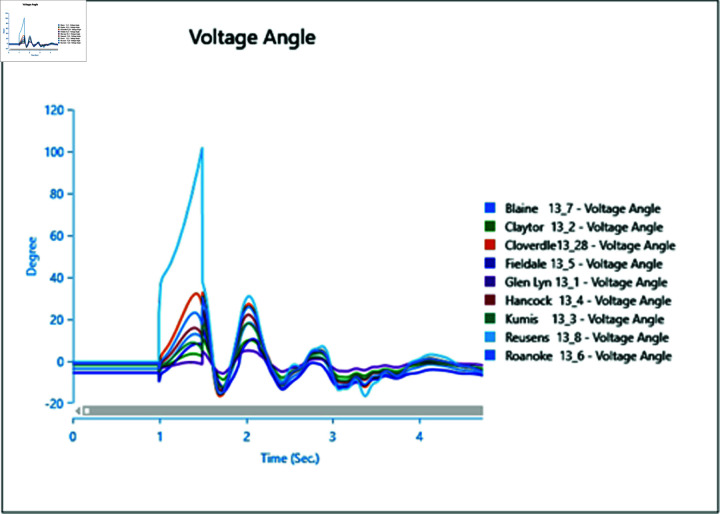
Case 5: Scenario 1 (voltage angle).

In Case 1, a synchronous generator alone is connected to the bus. When a fault occurs, there’s a noticeable change in the voltage profile, as indicated by Figs 20 and 21. However, once the fault is resolved, the system gradually returns to its rated values with some oscillations. In Case 2 when a wind turbine is added to the bus, contributing 50% of the generation capacity (45 MW), with the remaining provided by the synchronous generator, Figs 22 and 23 reveal fewer oscillations in the voltage phase shift, and adjacent bus voltage magnitudes stabilize similar to Case 1.

In Case 3, with a penetration level of 75% from the wind turbine and 25% from the synchronous generator, there’s no significant change in voltage profiles compared to Case 1 and Case 2 as shown in Figs 24 and 25, suggesting that integrating wind energy could yield similar impacts on voltage profiles during a fault, while being more cost-effective and environmentally friendly.

In Case 4, when PV generation is added at 50% capacity (45 MW) on the selected bus, the voltage profile remains stable, though the phase shift graph in Fig 26 indicates a longer stabilization time, with no drastic change in voltage amplitude.

Finally, in Case 5, with 75% of the generation coming from the PV array, the voltage profile fluctuates more, although it eventually stabilizes, with higher changes in voltage levels observed (Fig 28). However, there’s no significant change in phase angles compared to the previous case.

Overall, the integration of PV arrays and WTGs helps maintain stable voltage angle post-fault compared to when only a generator is integrated, with fewer oscillations. Among these, the presence of the Wind Turbine Type-III demonstrates more efficient bus voltage stabilization.

#### 4.2.2 Scenario 2

In Scenario 2, Load 8 connected to bus 13_8 is increased to 150%, prompting an analysis of the voltage and frequency at bus 13_8 alongside other buses. The simulation initializes the load impact at 1.0 second, with a total runtime of 5 seconds and analysis conducted for 0.5 seconds. Plots for analysis from Cases 1 through 5 are presented accordingly.

**Fig 30:**
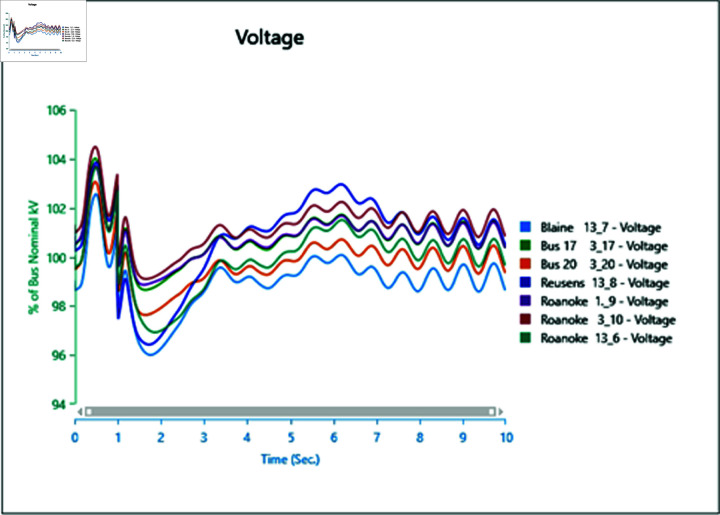
Case 1: Scenario 2 (voltages).

**Fig 31:**
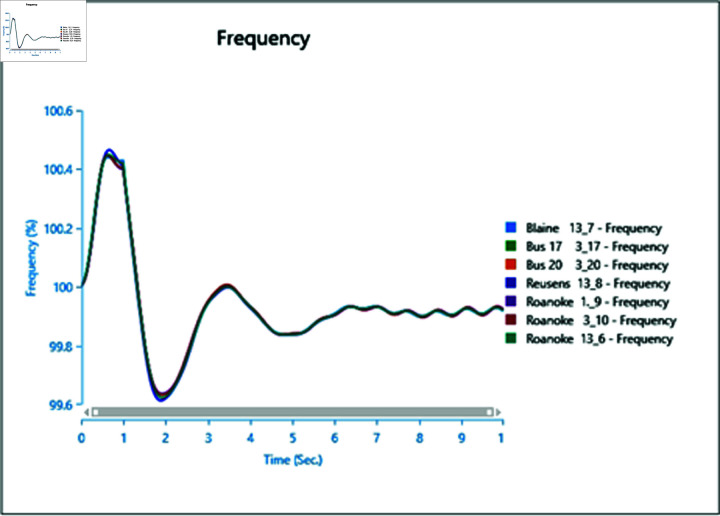
Case 1: Scenario 2 (voltage angle).

**Fig 32:**
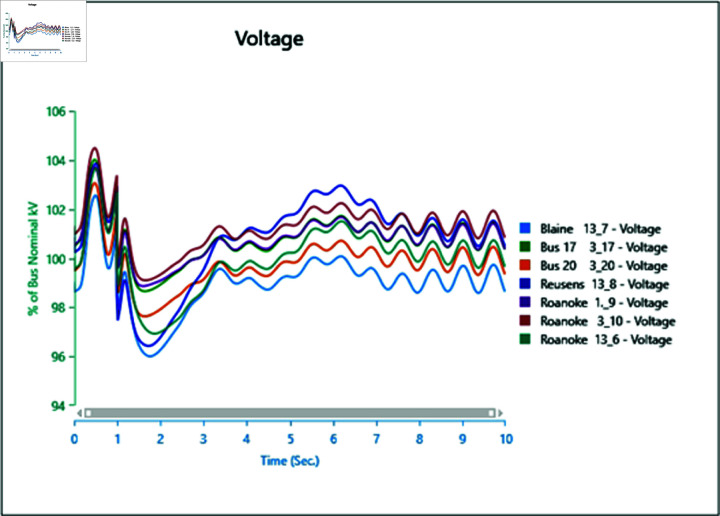
Case 2: Scenario 2 (voltages).

**Fig 33:**
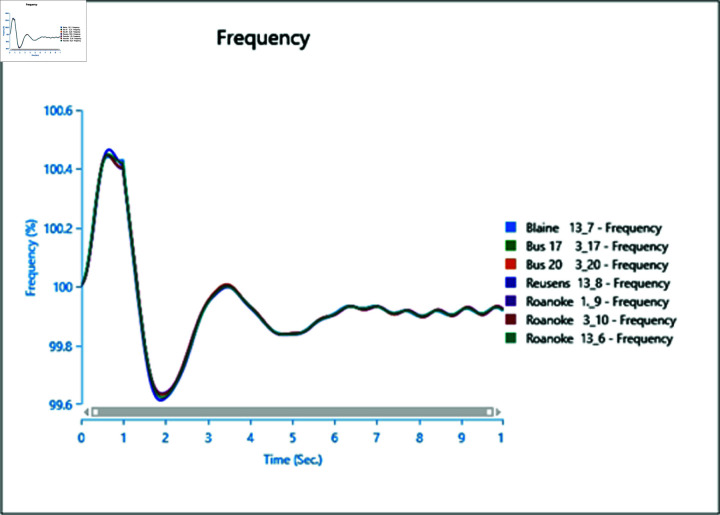
Case 2: Scenario 2 (voltage angle).

In Case 1, depicted in Figs 30 and 31, disturbances occur following the load impact at bus 13_8, with the synchronous generator unable to stabilize even after the load impact. Case 2 introduces changes in voltage oscillations and frequency (Figs 32 and 33) due to the addition of a wind turbine, although it does not fully address the load impact issue. However, with proper controller selection across the turbine, it may offer frequency support as an ancillary service (Fig 33).

**Fig 34:**
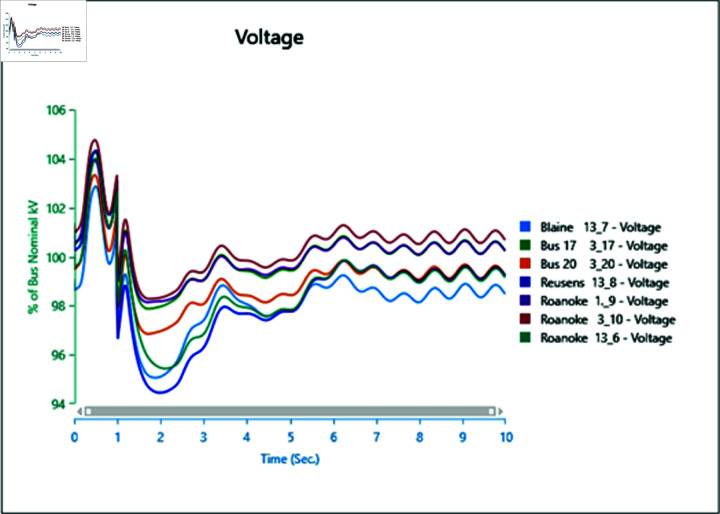
Case 3: Scenario 2 (voltages).

**Fig 35:**
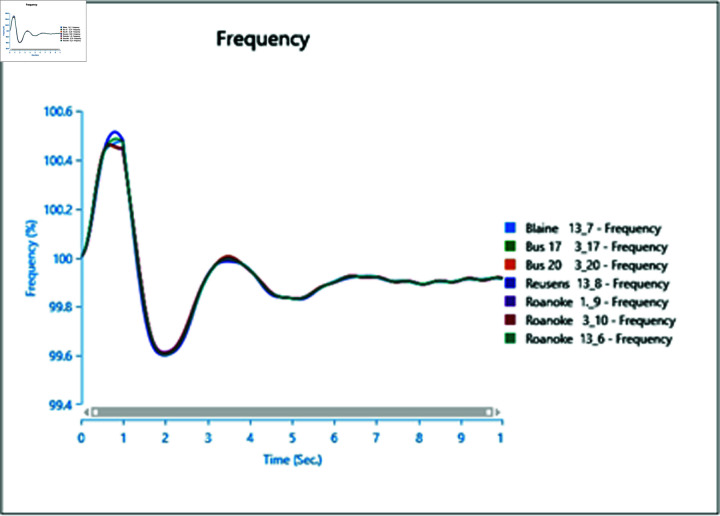
Case 3: Scenario 2 (voltage angle).

**Fig 36:**
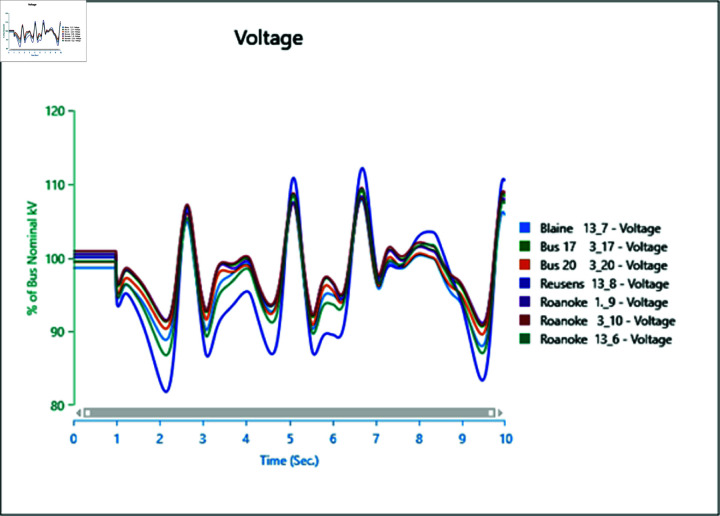
Case 4: Scenario 2 (voltages).

**Fig 37:**
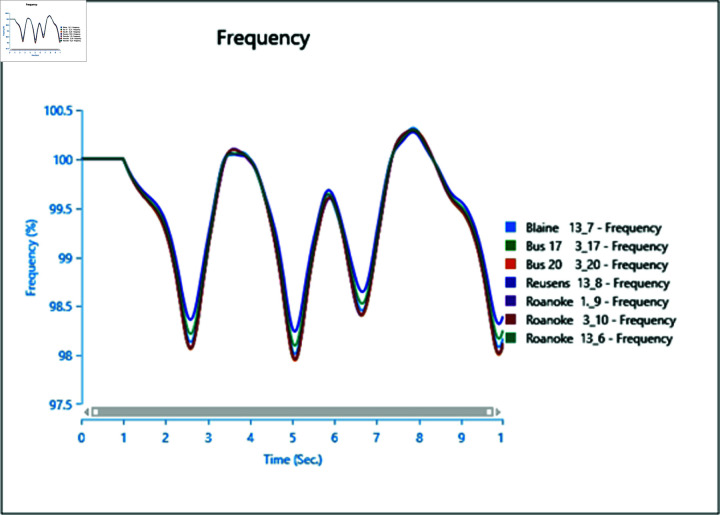
Case 4: Scenario 2 (voltage angle).

**Fig 38:**
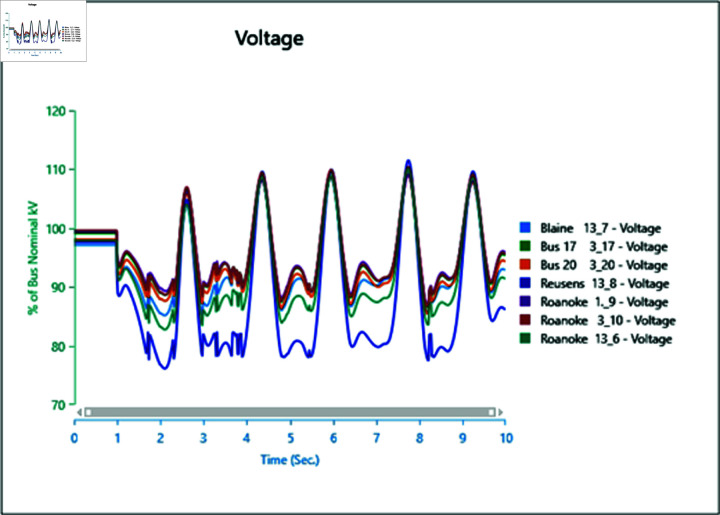
Case 5: Scenario 2 (voltages).

**Fig 39:**
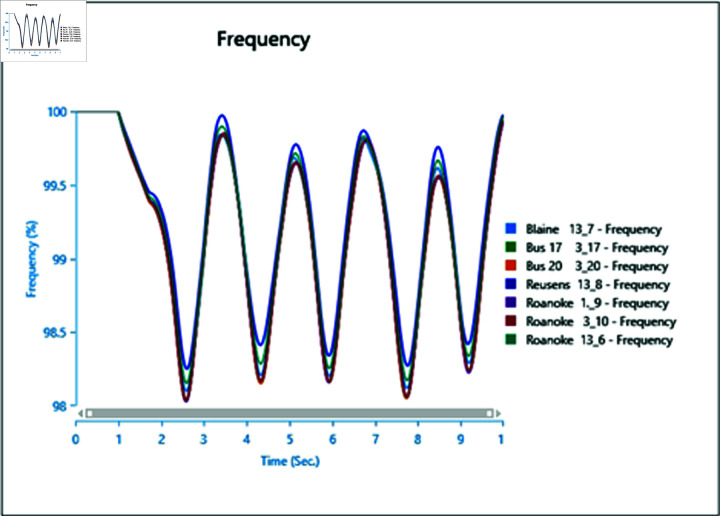
Case 5: Scenario 2 (voltage angle).

Increasing generation from the wind turbine to 75% (case 3) at the selected bus reduces oscillations in frequency output of adjacent buses (Fig 35), with minimal changes in voltage fluctuations (Fig 34). Addition of PV (case 4) at bus 13_8 leads to unstable voltages at adjacent buses, evident from Fig 36, while the frequency graph (Fig 37) indicates system desynchronization post-load impact. Implementing a generation controller for PV during peak sunlight can store excess power in a battery energy system, offering inertial support post-load impact. Further increasing PV penetration to 75% results in voltage and frequency profiles exceeding safe thresholds, as shown in Figs 38 and 39.

#### 4.2.3 Scenario 3

**Fig 40:**
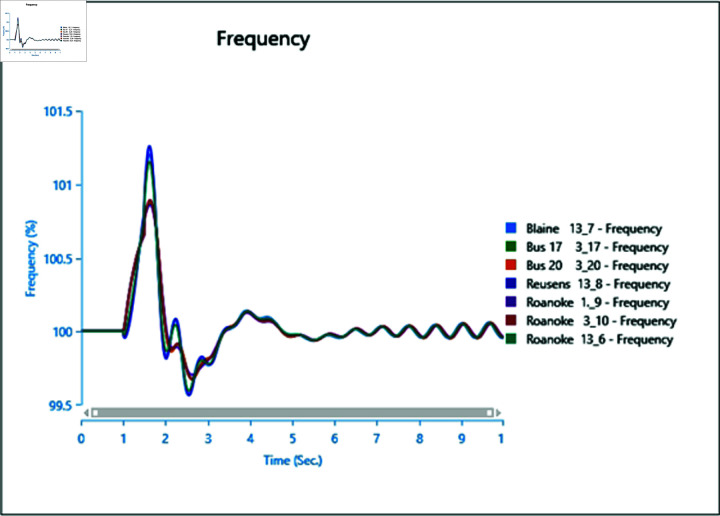
Case 1: Scenario 3 (frequency).

To assess the impact of the swing bus on the power system, a three-phase fault is induced on swing bus 13_1, and the frequency of the faulted bus is analysed alongside other buses. In Fig 40, the fault is initiated at swing bus 13_1 at 1 second, cleared after 0.5 seconds, leading to a frequency increase during the fault period, followed by a return to a steady state once the fault is cleared.

**Fig 41:**
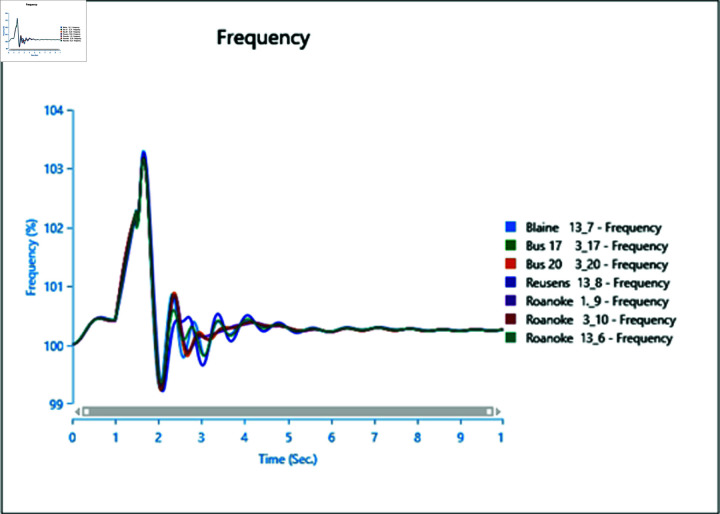
Case 2: Scenario 3 (frequency).

Introducing the same fault with the presence of a type-4 Wind Turbine results in slightly reduced oscillations post-clearance (Fig 41), indicating the potential for WTG to contribute to frequency stability as part of ancillary services.

**Fig 42:**
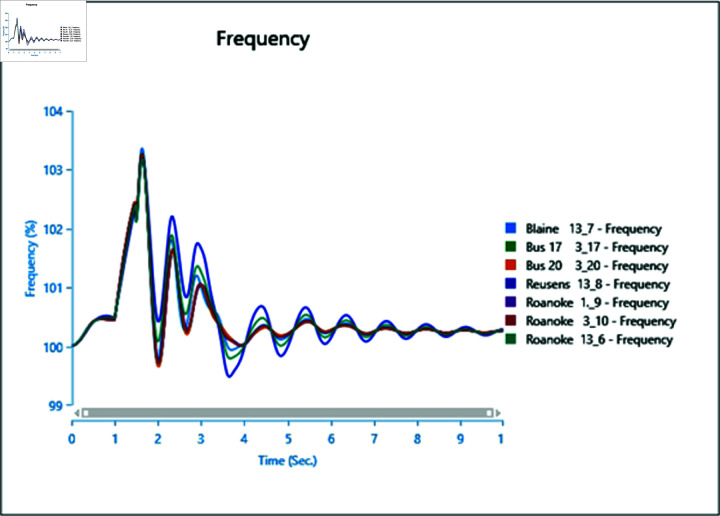
Case 3: Scenario 3 (frequency).

**Fig 43:**
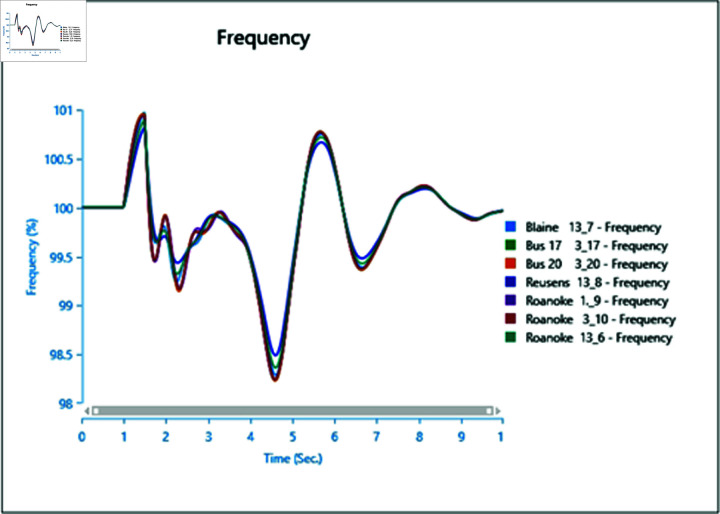
Case 4: Scenario 3 (frequency).

**Fig 44:**
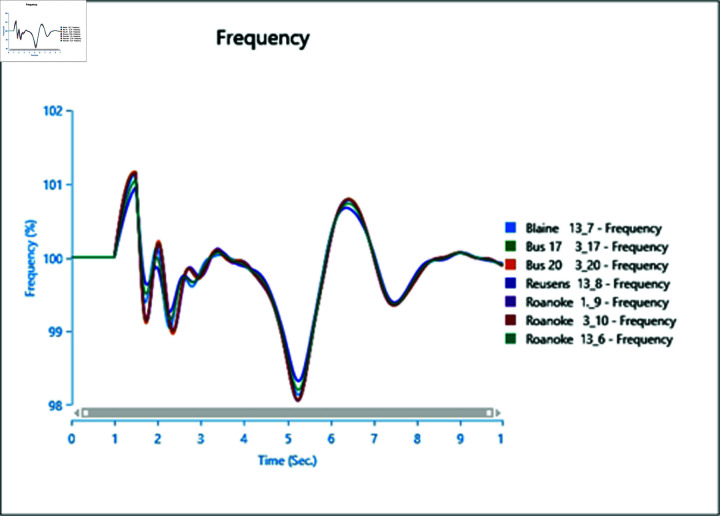
Case 5: Scenario 3 (frequency).

However, increasing wind turbine generation to 75% of the total connected generation at the selected bus does not significantly alter frequency oscillations after the fault (Fig 42), with increased oscillations observed instead. Upon adding PV arrays at bus 13_8 and inducing a three-phase fault at the swing bus, the system loses stability, highlighting a lack of frequency stability due to the inertia-less nature of PV arrays. Without a controller to provide support for frequency stability (Figs 43 and 44), further PV additions could exacerbate the situation.

### 4.3 Transient stability analysis for CGS and WTG

To apply the findings and utilize the data acquired from the controllers, a series of short circuit events were simulated at different bus bars. To explore the worst-case scenarios, specifically 3-phase short circuits were selected.

#### 4.3.1 Current stability of CGS and WTG

**Fig 45:**
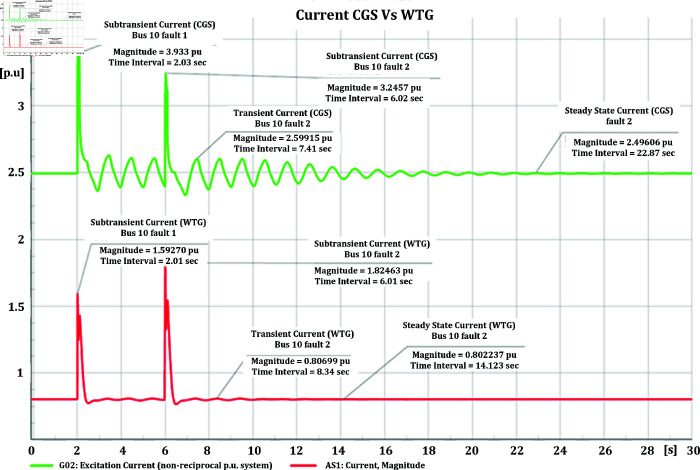
Current parameters CGS and WTG during short circuit event.

Fig 45 illustrates the current parameters at bus-10, where both the Conventional Generator System (CGS) and Wind Turbine Generator (WTG) are connected. Two types of short circuits, with impedances of 20+j5 ohm and 10+j2.5 ohm, were induced at this bus. The results clearly demonstrate differences between the CGS and WTG in terms of the fault currents’ magnitude and duration. Specifically, the sub transient current in CGS, initially at a magnitude of 3.24 pu, decreased to 1.648 pu, the transient current, initially at a magnitude of 2.59 pu, decreased to 0.75 pu, and the steady-state current, initially at a magnitude of 2.4 pu, decreased to 0.65 pu. While in WTG, sub transient current is initially at a magnitude of 1.824 pu, decreased to 0.826 pu, there is no significant sign of transient current as waveform get stable at 0.806 pu, and reaches to steady-state at 0.8022 pu.

#### 4.3.2 Frequency stability of CGS and WTG

**Fig 46:**
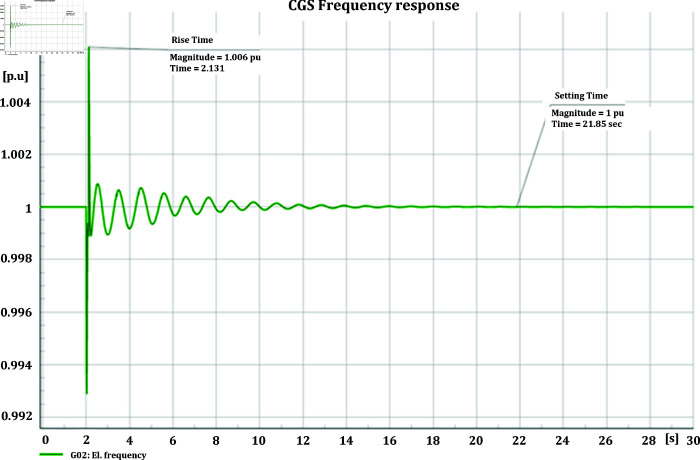
Frequency parameters CGS during short circuit event.

**Fig 47:**
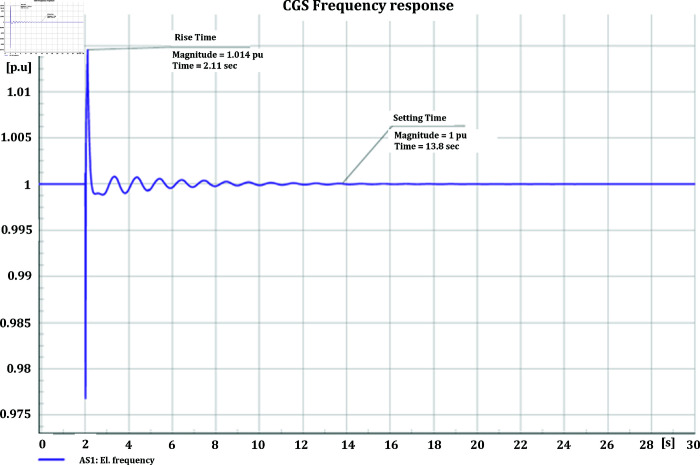
Frequency parameters WTG during short circuit event.

Figs 46 and 47 depict the frequency response of both the Conventional Generator System (CGS) and the Wind Turbine Generator (WTG) at the terminal of bus bar 10 during the occurrence of short circuit events with impedances of 20+j5 ohm and 10+j2.5 ohm. The results illustrate a reduction in frequency fluctuations in terms of both rise time and settling time. Specifically, the rise time for the CGS is 0.131 seconds, whereas for the WTG it is 0.11 seconds. Similarly, the settling time for the CGS is 21.85 seconds, whereas for the WTG it is 13.6 seconds.

#### 4.3.3 Voltage stability of CGS and WTG

**Fig 48:**
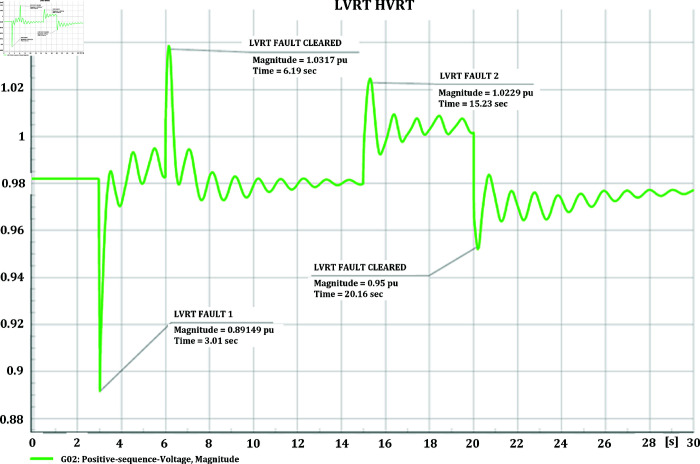
CGS LVRT and HVRT response during short circuit.

**Fig 49:**
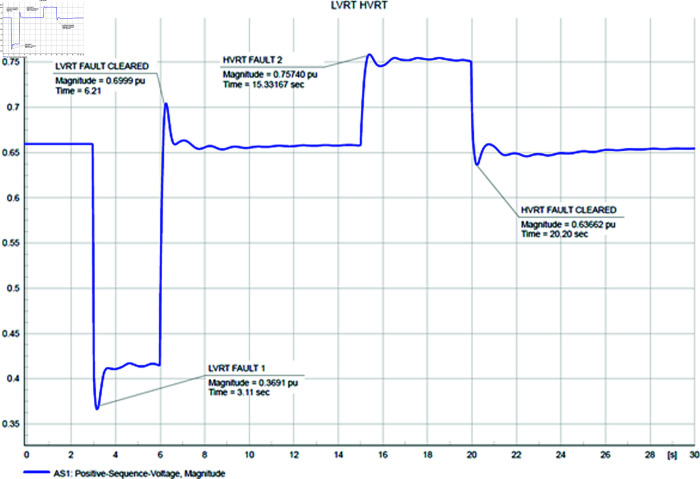
WTG LVRT and HVRT response during short circuit.

Figs 48 and 49 illustrate the voltage stability response of both the CGS and the WTG. For the Low Voltage Ride Through (LVRT) response, a 3-phase short circuit with an impedance of 50+j30 ohms is initiated at 3 seconds into the simulation and then cleared. Conversely, for the High Voltage Ride Through (HVRT) response, a 3-phase short circuit with a negative resistance of –100+j10 ohms is created. Typically, following a short circuit event, voltages experience a reduction before stabilizing. To simulate a High Voltage Ride Through scenario, a negative resistance fault is introduced, as such faults stem from a combination of mechanical and electrical issues within the network. The results demonstrate that in the CGS, during Low Voltage Ride Through (LVRT), the voltage remains constant at 1.03 pu initially, dropping to 0.86 pu due to transients induced by the fault. Upon fault clearance, additional transients occur before voltage stabilizes back to its normal operating level. Conversely, during High Voltage Ride Through (HVRT), the voltage increases from 1.03 pu to 1.05 pu, with some transient fluctuations, eventually stabilizing. In the case of the WTG, during LVRT, the voltage drops from a constant 0.6 pu to 0.38 pu immediately after the fault, and upon fault clearance, it swiftly returns to its normal condition. Meanwhile, during HVRT, the voltage rises from 0.6 pu to 0.76 pu, returning to its normal level immediately upon fault clearance.

### 4.4 Simulation results on impact of PF control and volt-VAR control method

**Fig 50:**
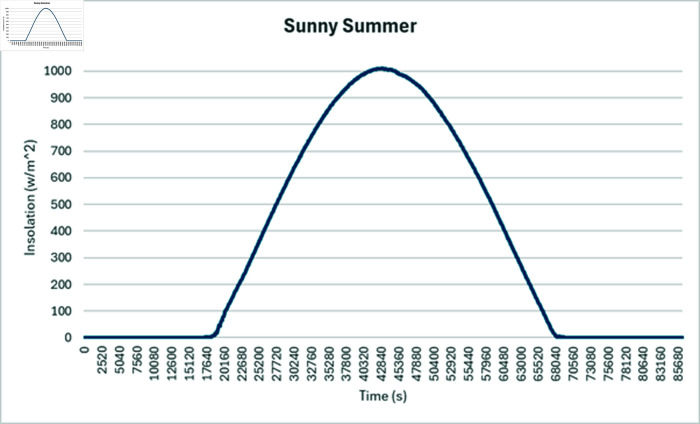
Insolation graph considered for sunny Summer.

**Fig 51:**
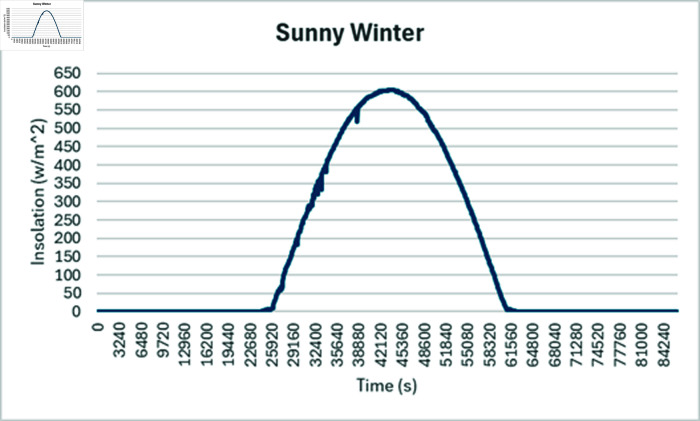
Insolation graph considered for sunny Winter.

**Fig 52:**
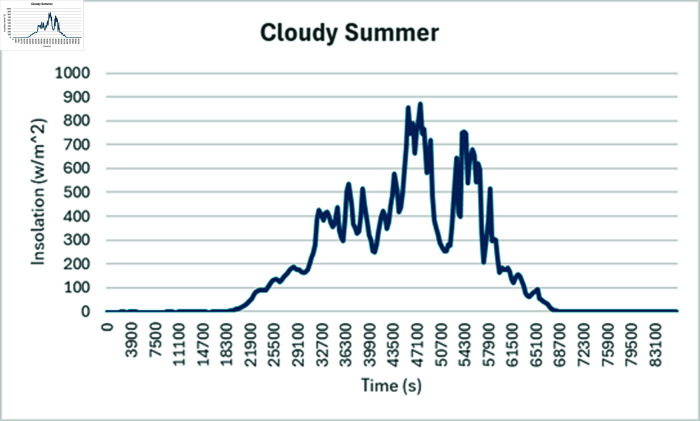
Insolation graph considered for cloudy Summer.

Simulation is conducted using CYME software, widely utilized in power flow analysis by various utility companies. This software enables the simulation of power systems with smart inverter control techniques, allowing for power variation during voltage fluctuations. In the PF control method, a fixed unity power factor is maintained to assess voltage changes under different PV penetration levels. Meanwhile, the Volt-Var control scheme prioritizes Var over watts, with voltage thresholds set at 90% as the lower limit and 110% as the upper limit. Three different weather conditions are observed to see the impact of PV system on distribution network, insolation profiles for all cases are shown in Figs 50–52.

#### 4.4.1 Case A

**Fig 53:**
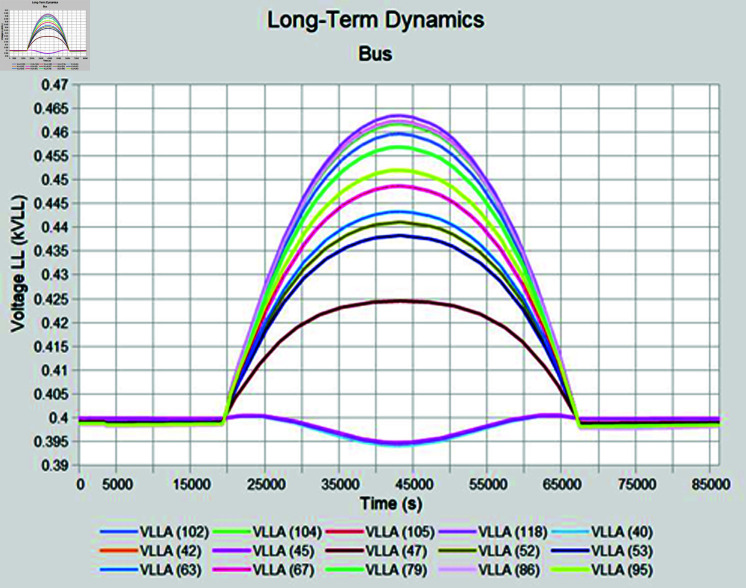
Voltage profile with PF control mode.

**Fig 54:**
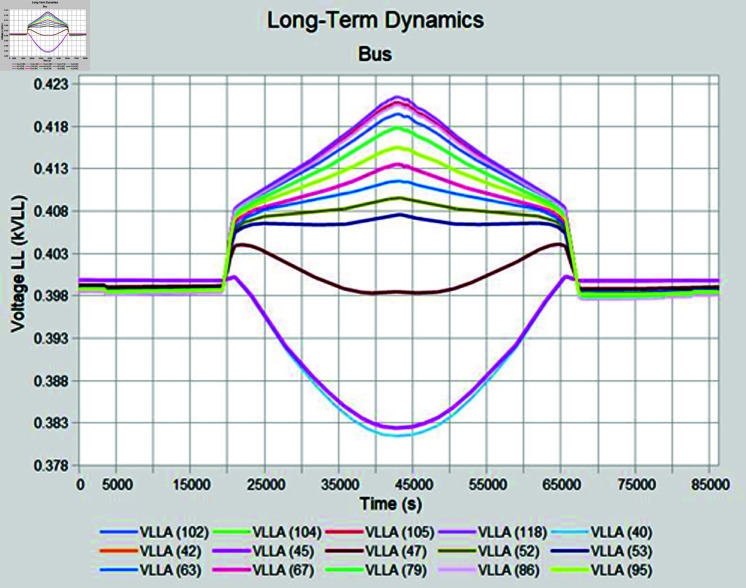
Voltage profile with Volt-Var control mode.

In Case A, sunny summer condition is considered where PV insolation will be high, smart inverter controls are applied to see the impact, PF control exhibits the lowest voltage of 395 volts while highest voltage recorded is 460 volts which is above the limits prescribed for safe operation as shown in Fig 53, Volt-Var control keeps voltages within limits but still one of the bus voltage has reached to lowest value of 370 volts while higher voltages are in limits as evident from Fig 54. Furthermore, Volt-Var control demonstrates the capability to limit PV generation during high insulation to prevent overvoltage, ensuring the point of common coupling voltage remains within acceptable limits.

#### 4.4.2 Case B

**Fig 55:**
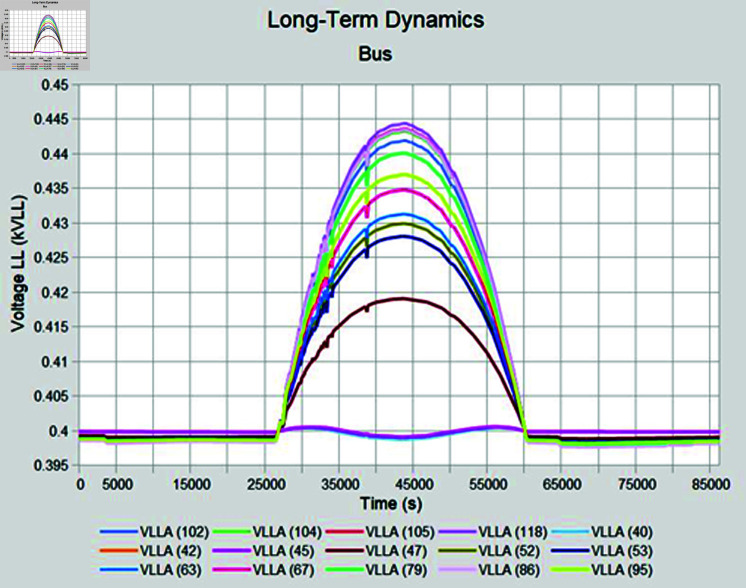
Voltage profile with PF control mode.

**Fig 56:**
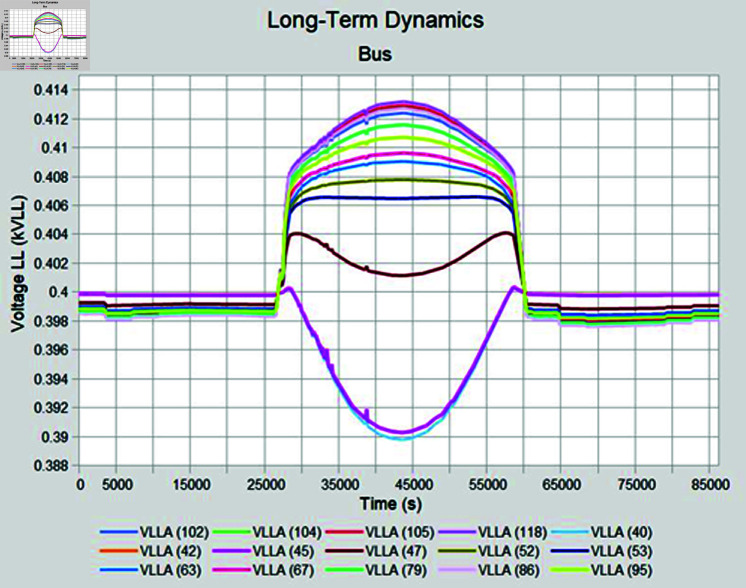
Voltage profile with Volt-Var control mode.

During sunny winter weather, with temperatures ranging from 20 to 33 degrees Celsius, there is typically a decrease in load compared to summer weather, at the same time PV insolation will be lower as well, under such condition, most of the buses maintain voltage profiles within acceptable limits for the PF control method. In sunny winter case voltage profile in PF control mode is reached to 465 Volts as maximum bus voltages while lowest bus voltages observed was 395 volts as shown in Fig 55. When Volt-Var control mode is applied upper voltages are in limits now while at the same time there will be PV output curtailment to keep grid voltages in limits. Figure 56 shows output voltages at different buses when Volt-Var mode is selected.

#### 4.4.3 Case C

**Fig 57:**
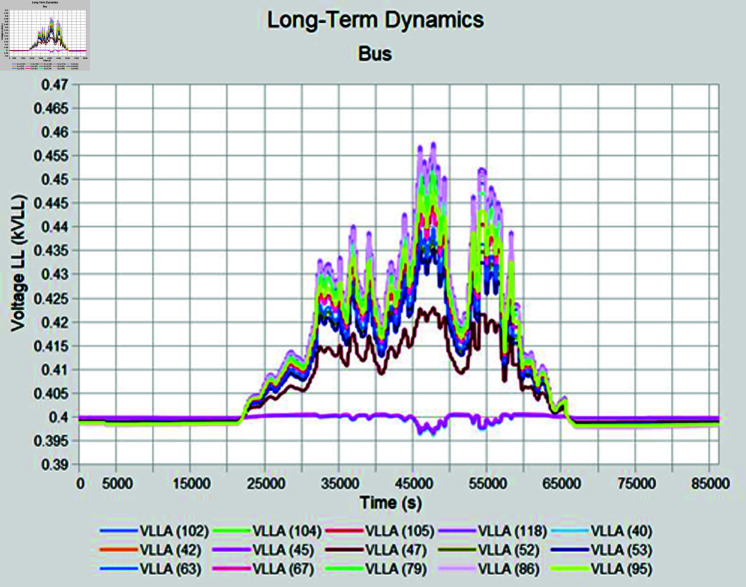
Voltage profile with PF control mode.

**Fig 58:**
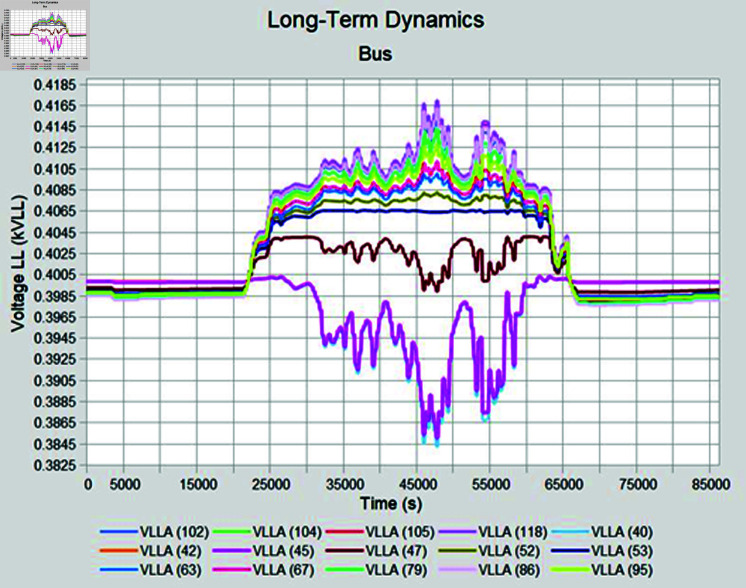
Voltage profile with Volt-Var control mode.

When cloudy summer case is selected, there are abrupt variations in the PV insolation due to cloudy weather which happens during monsoon season in Pakistan. Fixed PF mode is again not able to keep the voltages in acceptable range due to no PV curtailment; nearby feeders have to suffer overvoltage issue while operating in PF control mode (Fig 57). In Volt-Var control, as shown in Fig 58 results are better as now most of the buses are in acceptable voltage ranges except some buses who have a low voltage profile even before PV is installed so those buses are showing lower voltages even during Volt-Var control mode.

### 4.5 Results on SVCs control for medium transmission line

**Fig 59:**
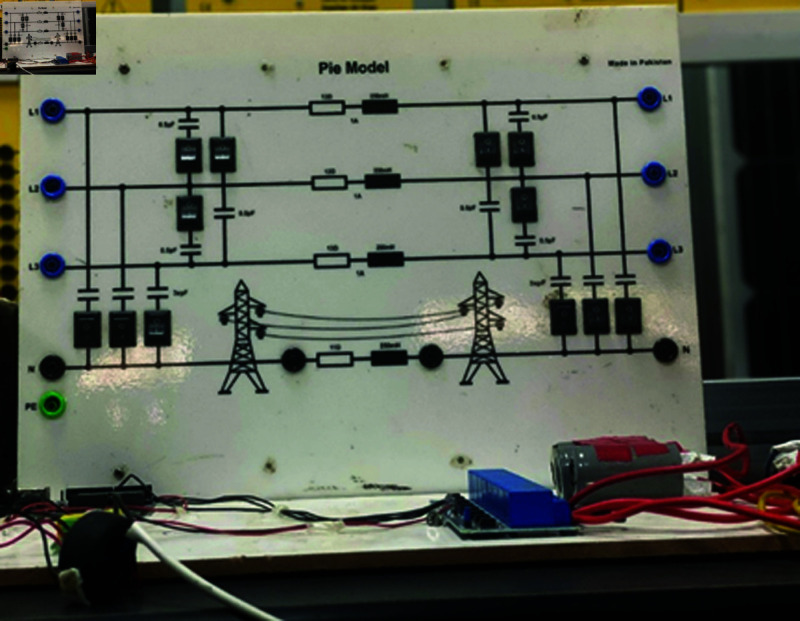
Indigeneously designed medium transmission line trainer.

This section discusses the results obtained from Static VAR control on medium transmission line. The hardware setup includes the indigenous-designed medium Transmission Line Trainer, the PZEM-004T energy meter, the Arduino microcontroller, and the 8-channel relay module. The medium Transmission Line Trainer replicates the characteristics of a medium transmission line, featuring various components such as inductive and resistive elements to simulate real-world impedance and electrical parameters. This hardware setup forms the basis of the static var compensator system, facilitating the monitoring of electrical parameters and control of the capacitor bank. A power meter has been integrated into the circuit to validate the results obtained. The system underwent testing with inductive and resistive loads connected in series, with additional validation through a power meter connected to the Arduino’s serial monitor. Fig 59 shows the complete hardware comprising of Transmission line trainer and SVCs control setup.

**Fig 60:**
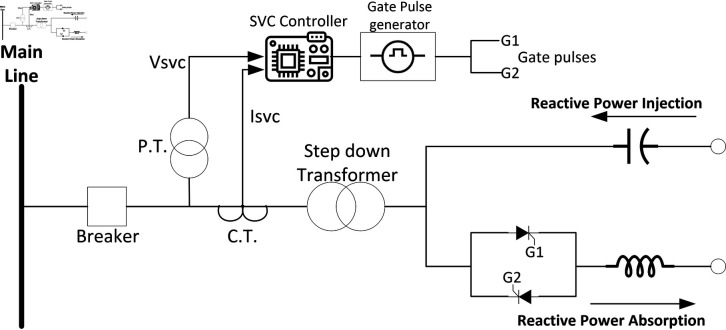
SVCs basic design.

**Fig 61:**
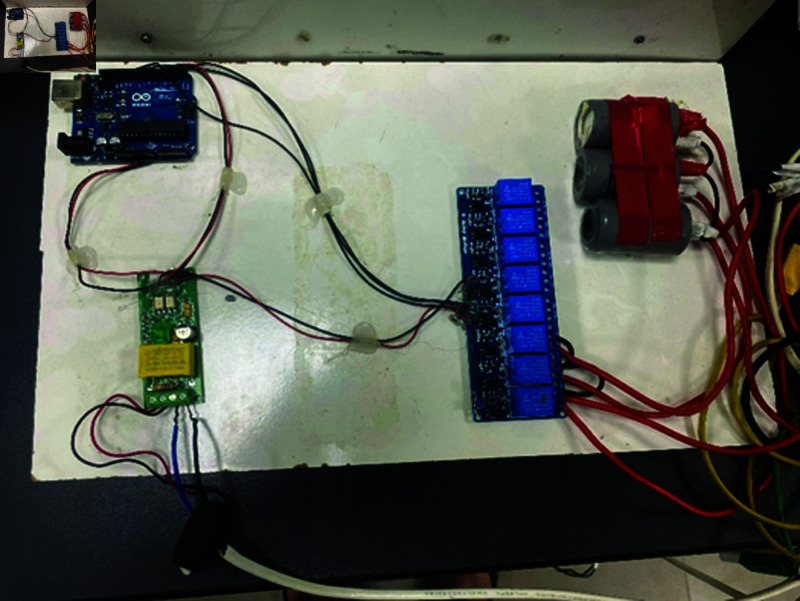
SVC control prototype.

**Fig 62:**
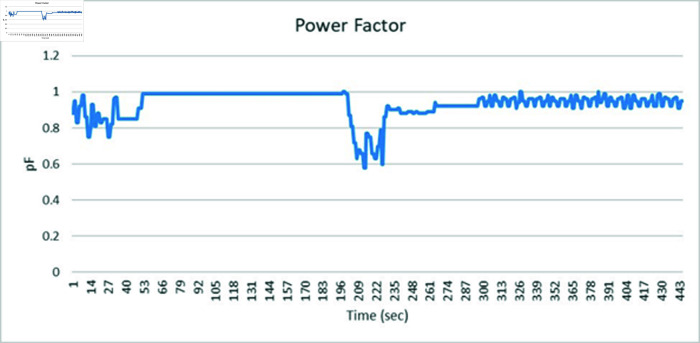
Power factor graph.

**Fig 63:**
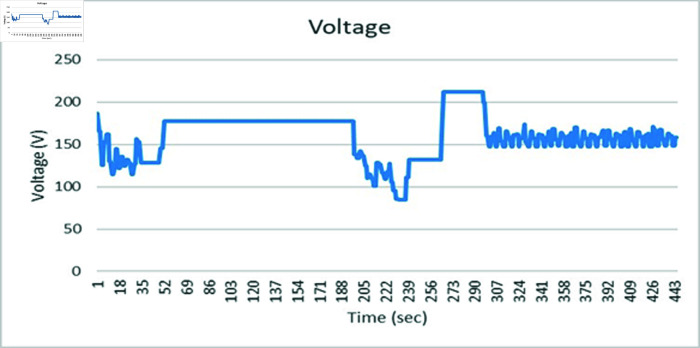
Voltage graph of medium transmission line.

**Fig 64:**
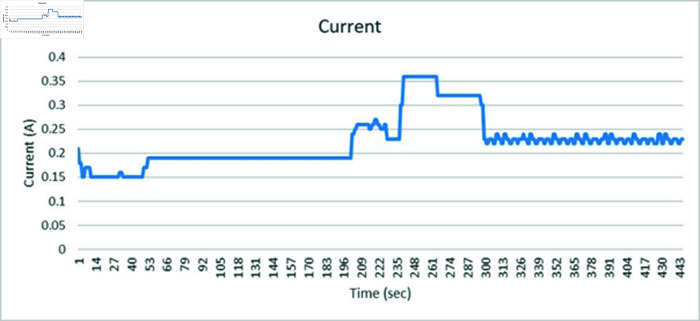
Current graph of medium transmission line.

**Fig 65:**
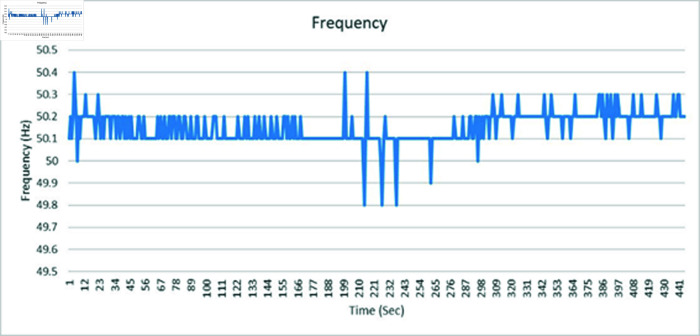
Frequency graph of medium transmission line.

Fig 60 is showing the circuit design of SVCs, SVCs system hardware is shown in Fig 61 which will be used to switch the relay module for the activation and deactivation of the capacitor bank. To evaluate the performance of this prototype, a time delay of 500 ms is considered to assess the switching speed of the relay module and the accuracy of this system. RL load banks are also utilized to induce variations in power factor and current, the main idea is to observe the impact of these load changes on the system’s behaviour. Initially, with a switching interval set at 500ms, RL loads are varied in seven steps, causing the power factor to drop below the threshold value of 1 to 0.65, as depicted in Fig 62. Subsequently, upon reaching the 500 ms mark, the relay module activated the capacitor bank, injecting reactive power into the transmission line and consequently improving the power factor back to 1. This process was repeated with additional inductive loads, necessitating a further injection of reactive power when the load became more inductive. Fig 62 underscores the capacitor bank’s role in injecting additional reactive power to restore the power factor to 1 following load variations. As evidenced by the fluctuations in Current and Voltage graphs as well (Figs 63 and 64). Another part of this section monitors the frequency continuously to observe the impact of reactive power compensation on system’s frequency as shown in Fig 65.

## Conclusion

This paper explores key areas for integrating renewable energy into smart grids: forecasting, stability, and power electronics. Accurate forecasting, using ARMA-based ANN models, allows proactive grid management, ensuring it can handle renewable generation. Wind turbines significantly enhance grid stability by improving voltage and frequency during faults, while solar panels have a minimal impact on voltage stability. However, combining wind and solar improves stability after faults, with wind being the stronger contributor. Power electronics, such as the Fast Local Voltage Controller and Wind Turbine Pitch Controllers, are crucial for maintaining grid stability. Future research should focus on fine-tuning these controllers for fault prediction and relay coordination to improve grid protection and reduce costs. Integrating renewables requires advancements in forecasting, power electronics, and grid management for a reliable, sustainable power grid.
